# Persistent inequalities in health care services utilisation in Brazil (1998–2019)

**DOI:** 10.1186/s12939-023-01828-3

**Published:** 2023-02-02

**Authors:** Maíra Coube, Zlatko Nikoloski, Matías Mrejen, Elias Mossialos

**Affiliations:** 1 Fundação Getúlio Vargas, São Paulo, Brazil; 2 Instituto de Estudos Para Políticas de Saúde (IEPS), São Paulo, Brazil; 3 Department of Health Policy, London School of Economics and Political Science, London, WC2A 2AE UK

**Keywords:** Inequalities, Utilisation, Curative Services, Preventative Services, Universal Health Coverage, Brazil, Health Care Services

## Abstract

**Background:**

One of the primary objectives of the Brazilian health care system is to improve the health and well-being of all citizens. Since the establishment of the Unified Health System/*Sistema Único de Saúde* (SUS) in 1988, Brazil has made strides towards reducing inequalities in health care services utilisation. However, there are currently no comprehensive and up-to-date studies focused on inequalities in both curative and preventive health care services utilisation.

**Methods:**

We evaluated data from the National Household Sample Survey and the Brazilian National Health Survey, which are two nationally representative studies that include findings from 1998, 2003, and 2008 and 2013 and 2019, respectively. We calculated Erreygers-corrected Concentration Indices (CInds) to evaluate the magnitude of socioeconomic-related inequalities associated with five indicators of health care services utilisation, including physician visits, hospital admissions, surgical procedures, Pap smears, and mammograms. The main factors associated with these inequalities were identified via a decomposition analysis of the calculated CInds.

**Results:**

While the results of our analysis revealed persistent inequalities in health care services utilisation that favour the wealthy, we found that the overall magnitude of these inequalities decreased over time. The largest inequalities were observed in the utilisation of preventive care services (Pap smears and mammograms) and services available in the poorest regions of the country. Except for admissions for labour and delivery, our findings revealed that wealthier individuals were more likely to utilise hospital services; this represents a change from findings reported in previous years. Private health insurance coverage and individual socioeconomic status are significantly associated with inequalities in health care services utilisation throughout Brazil.

**Conclusions:**

Collectively, our findings suggest that we must continue to monitor potential inequalities in health care service utilisation to determine whether Brazilian policy objectives focused on improved health outcomes for all will ultimately be achieved.

**Supplementary Information:**

The online version contains supplementary material available at 10.1186/s12939-023-01828-3.

## Background

Efforts to achieve timely and effective utilisation of health care services present an ongoing challenge to the goal of providing universal health care coverage to those living in low- and middle-income countries [1]. Both financial and non-financial barriers can impede the appropriate use of these services and may lead to poorer overall health outcomes [2–4]. Moreover, there are substantial inequalities regarding access to and utilisation of health care services even in countries that provide universal health care coverage [3]. In many cases, the most economically-advantaged members of the population utilise the vast majority of health care services in a given region. Inequalities are particularly evident with respect to the use of preventive health care services [5] and can be observed in both high-income countries that provide universal health care coverage as well as in middle-income countries [5, 6]. Inequalities in health care services utilisation that are skewed towards those of higher socioeconomic status frequently lead to substantial differences in overall health outcomes. To address these concerns, the evolution of health care services utilisation needs to be monitored. This is an essential first step towards the design of targeted policies that focus on reducing health care and outcomes disparities [2, 3].

Brazil presents a unique setting in which inequalities of health care services utilisation might be analysed. Brazil currently has one of the highest levels of economic inequality worldwide (Gini index of 48.9 in 2020) [7]. Likewise, although Brazil supports a single-payer, publicly funded national health service known as the Unified Health System/*Sistema Único de Saúde* (SUS), over half of the total expenditure on health care comes from private sources [8].

The SUS is a tax-based, non-contributory public health care system that provides services at all levels that are free of charge at the point of delivery. The system has achieved significant gains over the past 30 years; improvements in both coverage and access to health care services have resulted in better health outcomes overall. [9] A central feature of the national health service in Brazil is primary health care, mainly the Family Health Strategy (FHS) which covers > 60% of the population according to the coverage parameters defined by the Ministry of Health. One Family Health Team which includes a nurse and community health care workers is expected to provide care for approximately 3450 individuals [9]. The availability of primary health care services has clearly improved the health of the Brazilian population [10, 11] and has contributed to a reduction in health inequalities [12]. However, access to specialised care remains a major challenge in the public health care sector and long waiting times persist [8, 13]. Furthermore, while SUS provides universal health coverage, 28.5% of the population (primarily individuals with higher incomes who are formally employed and residing in urban centres) are also covered by one or more private health insurance schemes [14].

The highly fragmented nature of the current health care system and persistent regional disparities remain major barriers to more equitable access to health care services throughout Brazil [15]. Brazil has large geographical variations with unequal distribution of infrastructure, human resources, access to medications, and coverage by national health care programmes [16–19]. Variations in the supply of health care resources coupled with the two-tiered (public *versus* private) financing system has led to disparities in both access to and quality of health care delivered as well as protection from undue financial risk [15, 20]. Results from previous studies suggest that the limited availability of health care providers, infrastructure, and medications in underserved areas remains a major challenge [11, 15–19]. Likewise, the financial burdens associated with seeking care (e.g., transportation costs) represent an additional barrier that prevents the appropriate utilisation of health care services [21, 22].

Existing evidence suggests that the inequalities in health care services utilisation largely reflect the overall socioeconomic inequalities in Brazil [20, 23–27]. Although results from previous studies revealed a gradual improvement in reducing the inequalities in health care services utilisation between 1998 and 2008, the existence of private health insurance schemes has been identified as one of the largest contributors to the persistence of this problem. Most of these larger published studies cover only this earlier decade [20, 23–25]. By contrast, studies that include the more recent data (2013–2019), have focused on outcomes associated with specific health care services (e.g., preventive care) [26] or specific segments of the population (e.g., the elderly) [27].

The goal of this study is to evaluate socioeconomic inequalities and their role in the differential utilisation of health care services in Brazil from 1998 through 2019. Our study includes both past as well as more recent information focused on the utilisation of both preventive and treatment services. We also evaluated the role of private health insurance as well as the known socioeconomic and geographical disparities on health care services utilisation in Brazil.

## Methods

### Dataset and sample

We examined data from two nationally-representative cross-sectional household surveys performed in Brazil. We reviewed findings from the National Household Sample Survey PNAD (*Pesquisa Nacional por Amostra de Domicílios*), which included a Health Supplement in 1998, 2003, and 2008. We also evaluated results from the Brazilian National Health Survey PNS (*Pesquisa Nacional de Saúde*) which was conducted in 2013 and 2019. Both surveys were conducted by the Brazilian Institute of Geography and Statistics (*Instituto Brasileiro de Geografia e Estatística* [IBGE]) in partnership with the Ministry of Health using a complex probabilistic sample design that was clustered in three stages. Microdata provided by IBGE included all the information needed to account for the sampling design, including weights adjusted for non-response rates and population projections [14, 28, 29].

The survey sample was designed to be representative at the national level as well as within the five major regions of Brazil (i.e., South, Southeast, North, Northeast, and Midwest). To maintain sample consistency over time, we excluded rural areas from the North because these regions were not included in national household surveys until 2004 [28]. The structure of the questionnaire in both surveys was adjusted to be representative of the same populations while allowing for the analysis of temporal trends. Both the PNAD and the PNS collected a wide array of socioeconomic characteristics, information on health status, and the utilisation of health care services by all members of the households that were sampled. Questions focused on lifestyle as well as diagnosis and treatment of chronic diseases were answered by a single, randomly-selected household member who was at least 18 years of age in 2013 and at least 15 years of age in 2019. In both surveys, there is one respondent per household answering questions about him/herself as well as others that pertained to other members of the household. Data are made publicly available at the individual level. The survey contains a comprehensive set of questions that facilitates the implementation of the Andersen framework for analysing access to health care services [30]. The Andersen framework proposes that the access to selected health care services (e.g., outpatient and inpatient) could be explained by an extensive set of factors that can be categorised into three major groups, including (i) predisposing factors such as age or gender (e.g., older patients may require more health care visits than younger ones), (ii) enabling factors such as income or education (e.g., wealthier patients may have better access to health care services), and (iii) need factors such as overall health status (e.g., chronically ill patients may utilise more health care services). This theoretical framework is explained further in the methods section below. To maintain consistent comparisons over time, our sample for this analysis was restricted to responses from individuals who were 18 years of age and older.

### Variables associated with health care services utilisation

We computed five measures of health care services utilisation that fall within three levels of care, including preventive care (Pap smears and mammograms), outpatient care (physician visits during the past year), and inpatient care (hospitalisations and surgical procedures during the past year). The two outcome variables representing preventive care were calculated by restricting the sample to eligible women based on age ranges and screening periods for each test as indicated by guidelines from the Brazilian Ministry of Health. These include Pap smears once every three years for women between 25 and 59 years of age, and mammograms performed once every two years in women between 50 and 69 years of age [31, 32]. These two variables were not computed in the 1998 survey; data on these procedures were collected from 2003 onwards. As our study focuses only on need-based utilisation of inpatient care, we have excluded hospital admissions for labour and delivery, including those for vaginal births and Caesarean sections. However, these findings are included in the Supplementary analysis. All questions were included in all rounds of the surveys. Additional details are included in Additional file 1 Appendix 1.

### Methodology

We computed the Concentration Indices (CInds) to examine the magnitude of socioeconomic-related inequalities and their relationship to health care services utilisation. This approach was coupled with a decomposition analysis of the CInds to assess the extent to which various factors were associated with the inequalities in utilisation.

First, we plotted concentration curves with the cumulative percentage of utilisation variables on the y-axis and the cumulative percentage of the population ranked by household income per capita on the x-axis. The associated CInd is a summary measure that equals twice the area between the concentration curve and the line of equality (the 45° line). Thus, the CInd is a value between -1 and 1 with negative values indicative of a pro-poverty skew and positive values representing a skew toward wealth. The CInd is then computed as [33]

1 $$C\left(h\right)=\frac{1 }{n}{\sum }_{i=1}^{n}\left[\left(\frac{ {h}_{i}}{\overline{h} } \right)(2{R}_{i}^{y}-1)\right]$$

where $C\left(h\right)$ is the CInd of variable h (health care services utilisation), ${h}_{i}$ is the value of $h$ for individual $i$, $\overline{h }$ is the sample mean of $h$, $n$ is the sample size, and ${R}_{i}^{y}= {n}^{-1} \left(i-0.5\right)$ is the fractional rank of individual $i$ ordering of the sample according to household income per capita (lowest to the highest). We recognise certain shortcomings associated with the standard calculation of CInd, for example, the ‘bounds issue’ for bivariate variables [34, 35]. In other words, two regions with equal CInds that have different mean rates of utilisation of a given service will be interpreted as reflecting different levels of inequality because the mean of the distribution places bounds on the possible values of the CInd [36]. Thus, when quantifying the magnitude of socioeconomic-related inequality and its association with health care services utilisation, we applied the correction for binary variables suggested by Erreygers [34]. The Erreygers-corrected CInd is computed as:

2 $$E\left(h\right)=\left(\frac{4\overline{h}}{{h }_{max}- {h}_{min} }\right) C\left(h\right)$$

The Erreygers-corrected CInd, or *E(h)*, derived from this analysis satisfied the four properties that are considered to be desirable when the variable of interest is binary, including (1) small transfers of health from richer to poorer individuals (or vice versa) translates into a “pro-poor” change in the index (or vice versa), (2) inequality indicators for utilisation and non-utilisation of health care services are mirror images of one another, (3) an equal incremental change in the health of all individuals has no impact on the CInd, and (4) linear transformation of the health variable has no impact on the value of the CInd. Additional details related to this analysis are available in Additional file 1 Appendix 1 [34].

There are a few compelling reasons that explain why we have opted for the Erreygers-corrected CInd. First, the correction of this index acknowledges the boundedness of the health variable (in this particular instance, the utilisation of a specific health care service). Moreover, as previously discussed, the type of preferred index will depend on a researcher’s value judgment [35]. Thus, the research objectives and questions guide what index or normalisation scheme is chosen by the researchers, despite the differences in interpretation [36]. As we are primarily interested in absolute inequalities, the selection of Erreygers-corrected CInd is justified. Finally, the Erreygers-corrected CInd satisfies three major conditions, including linearity, convergence, and monotonicity. These three properties facilitate data interpretation and will help us to anticipate the impact of health changes on the index [36].

We coupled the CInd analysis with the usual decomposition analysis to identify one or more factors associated with the observed inequalities in health care services utilisation [34, 37]. Building on the theoretical framework proposed by Andersen [30], the range of independent variables used in the decomposition analysis was divided into three major groups to capture predisposing (e.g., age, gender, employment, race), enabling (e.g., socioeconomic standing, private health insurance, regional disparities in infrastructure), and need factors (e.g., overall health status and/or carrying a diagnosis of one or more non-communicable diseases). Further details of the entire set of these variables are included in Additional file 1 Appendix 2.

Decomposition of the CInd may reveal an association between the independent variables and income-related inequalities in health distributed over the entire population [33]. This analysis provides more detailed information and identifies areas that may benefit from specific policy interventions. Decomposition analysis relies on the assumption of a linear model that links the health variable h (the utilisation variables) to one or more contributing factors. As our dependent variable is binary, we relied on methodology for the decomposition analysis that featured a probit model with partial effects as depicted in Eq. 3

3 $$E\left({y}_{i}|{x}_{i}\right)=G \left({\sum }_{j}{\beta }_{j}{x}_{i}^{j}\right)$$

where *G* represents the functional form for a nonlinear model. As proposed by van Doorslaer et al. [38] we restored the mechanics of the decomposition framework by replacing ${\beta }_{j}$ in the equation with the ${\beta }_{j}^{m}$ parameters, where ${\beta }_{j}^{m}$ represents the partial effects of $x$ (the determinants of $y$) for each individual $i$ with characteristics $k$ and ${\mu }_{i}$ and is the error term in the linear approximation of the non-linear model, expressed by Eq. 4:

4 $${y}_{i}= {\sum }_{j}{\beta }_{j}^{m}{x}_{i}^{j}+ {\mu }_{i}$$

For example, the partial effect of female gender is calculated as the mean of ${\beta }_{j}^{m}$ for all female participants. This calculation captures the fact that there may be characteristics other than sex that differentiate the female population from the whole. Finally, we imputed the beta (β) calculated as described above to compute the Erreygers-corrected CInd as follows:

5 $$E\left(h\right)=4\left[\sum_{j=1}^{k}{\beta }_{j}^{m}GC\left({x}_{j}\right)+GC({e}_{i})\right]$$

Thus, the contribution of each explanatory variable ${x}_{j}$ is the result of the product of the sensitivity of health with respect to that factor, ${\beta }_{j}^{m}$, and the degree of income-related inequality in the distribution of that factor, i.e., the generalized concentration index $GC\left({x}_{j}\right)= \overline{{x }_{j}} \times C({x}_{j})$. All coefficients ${\beta }_{j}^{m}$ were partial effects estimated using the linear approximation of the non-linear relationship between the covariates and our dependent variables as presented in Eq. 4. We used a decomposition technique to evaluate the factors contributing to socioeconomic inequalities in health care services utilisation in 2019. It is important to note that CInd calculates the utilisation of a given health care service in relation to income. Thus, all analyses presented here are based on how CInd affects the use of that particular health care service in relation to income. Further details and a description of the entire set of covariates ${x}_{j}$ are provided in Additional file 1 Appendix 2.

## Results

The results shown in Table 1 summarise the five variables of interest, including (1) any visits to a physician during the past year; (2) any hospitalisations during the past year, excluding those for labour and delivery; (3) any surgical procedures during the past year; (4) use of Pap smears during the past three years by women between 25 and 59 years of age; and (5) use of mammograms during the past two years by women between 50 and 69 years of age. In 2019, the health care services used most frequently were physician visits (80.8%; 95% [Confidence Interval] CI, 80.4%–81.3%). By contrast, only 6.5% (95% CI, 6.2%–6.9%) of the adult population (18 years of age and older) reported being hospitalised for reasons other than labour and delivery. Interestingly, when hospital admissions for labour and delivery were included in this calculation, 7.6% of the population reported at least one hospitalisation during the past year (95% CI, 7.2%–7.9%; see Additional file 1 Appendix Table A1). Likewise, only 3.0% of the population (95% CI, 2.7%–3.3%) underwent a surgical procedure during the past year.

**Table 1:** Summary statistics for dependent variables

	**1998** (***N*** = 215,283)			**2003** (***N*** = 252,1087)			**2008** (***N*** = 263,902)			**2013** (***N*** = 57,195)			**2019** (***N*** = 83,927)		
**Variable**	n	%	95% CI	n	%	95% CI	n	%	95% CI	n	%	95% CI	n	%	95% CI
Any doctor visit in the past year	123,688	57.4	(56.9–57.9)	162,363	64.4	(64–64.8)	184,467	69.9	(69.5–70.2)	42,496	74.3	(73.6–74.9)	67,813	80.8	(80.4–81.3)
Hospitalisation in the past year (excluding labour and delivery)	14,006	6.5	(6.3–6.6)	16,387	6.5	(6.4–6.7)	17,681	6.7	(6.6–6.9)	3,432	6.0	(5.7–6.3)	5,455	6.5	(6.2–6.9)
Any surgery in the last year	4,310	2.0	(1.9–2.1)	5,799	2.3	(2.2–2.3)	6,070	2.3	(2.3–2.4)	1,373	2.4	(2.2–2.6)	2,518	3.0	(2.7–3.3)
Use of Pap smears in the past three years (women aged 25–59 years)				63,606	73.6	(73.1–74.1)	73,228	79.1	(78.7–79.6)	17,809	79.9	(78.9–80.8)	23,684	82.3	(81.6–83)
Use of mammograms in the past two years (women aged 50–69 years)				12,151	44.8	(44.1–45.6)	16,953	53.2	(52.5–53.9)	4,546	54.3	(52.5–56.2)	8,464	58.3	(56.9–59.6)

The findings presented include the mean prevalence for all outcome variables, including the number of observations (n), percentage (%), and 95% confidence intervals (CIs). ***N*** refers to the total sample of the adult population 18 years and older corresponding to the sample size of the first three outcomes. The total sample of the outcome variables including “use of Pap smear in the past three years” and “use of mammogram in the past two years” includes only women who are eligible for these screening tests based on guidelines from the Brazilian Ministry of Health. The number of observations for women 25–59 years of age include *n* = 86,421 (2003), *n* = 92,577 (2008), *n* = 22,289 (2013), and *n* = 28,778 (2019). The number of observations for women 50–69 years of age include *n* = 27,123 (2003), *n* = 31,866 (2008), *n* = 8,372 (2013), and *n* = 14,518 (2019). The outcome variable listed as “hospitalisation in the last year” excludes admissions for labour and delivery, both for a vaginal birth and Caesarean sections

*Sources:* PNAD 1998, 2003, and 2008, PNS 2013 and 2019, and the authors’ calculations

With respect to preventive screening services, 58.3% (95% CI, 56.9%–59.6%) of those eligible underwent a mammogram while 82.3% (95% CI, 81.6%–83%) had a Pap smear. Over time, the utilisation of health care services (defined as the share of respondents that have used a specific service) has increased (Table 1). The largest increase in health care services utilisation between 1998 and 2019 was in the frequency of physician visits at 23.4 percentage points. By contrast, the utilisation of inpatient services remained virtually unchanged during this period. The use of preventive services also increased between 2003 and 2019 by 8.7 and 13.5 percentage points for Pap smears and mammograms, respectively. The remaining variables are summarised and discussed in Additional file 1 Appendix Table A2 and Table A1.

Disaggregation of these results by socioeconomic status provides some explanation for our findings (Additional file 1 Appendix Figure A1). First, the number of physician visits, surgical procedures, and use of preventive services per capita was highest among the respondents in the highest income quintiles; this pattern has remained stable over time. By contrast, the prevalence of hospitalisations across income quintiles has changed over time. While those in the lowest income quintile reported fewer hospitalisations in 2019 compared to 1998, the prevalence of hospitalisation among those in the most wealthy segments of the population increased slightly during the same period.

Erreygers-corrected CInds and the concentration curves for each of the five variables of interest are shown in Table 2 and Additional file 1 Appendix Figure A2, respectively (as well as Table A3 for the hospitalization variables). As noted above, a CInd with a negative coefficient represents a situation in which inequalities are more prevalent among those of lower socioeconomic status (i.e., pro-poor). By contrast, a positive coefficient represents an unequal distribution of the outcome variable in a direction that favours those of higher socioeconomic status (i.e., pro-rich). Our analysis of the data collected in 2019 revealed that all health care service utilisation variables were skewed significantly in the pro-rich direction, with Erreygers-corrected CInds of 0.114 (95% CI, 0.103–0.124) for physician visits, 0.010 (95% CI, 0.004–0.016) for hospitalisations, 0.016 (95% CI, 0.012–0.021) for surgical procedures, 0.137 (95% CI, 0.121–0.152) for Pap smears, and 0.265 (95% CI, 0.235–0.294) for mammograms.

**Table 2:** Erreygers-corrected CInds for health care utilisation variables

	**1998**		**2003**		**2008**		**2013**		**2019**	
**Variables**	CInd	95% CI	CInd	95% CI	CInd	95% CI	CInd	95% CI	CInd	95% CI
Any doctor visit in the past year	0.121***	(0.111–0.130)	0.136***	(0.128–0.143)	0.105***	(0.099–0.111)	0.135***	(0.122–0.149)	0.114***	(0.103–0.124)
Hospitalisation in the past year (excluding labour and delivery)	-0.004***	(-0.007–0.001)	0.004***	(0.001–0.007)	0.003*	(-0.000–0.005)	0.003	(-0.005–0.010)	0.010***	(0.004–0.016)
Any surgery in the past year	0.011***	(0.010–0.013)	0.014***	(0.012–0.015)	0.013***	(0.011–0.015)	0.011***	(0.006–0.016)	0.016***	(0.012–0.021)
Use of Pap smears in the past three years (women aged 25–59 years)			0.201***	(0.191–0.210)	0.145***	(0.136–0.154)	0.156***	(0.135–0.177)	0.137***	(0.121–0.152)
Use of mammograms in the past two years (women aged 50–69 years)			0.410***	(0.395–0.424)	0.348***	(0.334–0.361)	0.359***	(0.321–0.396)	0.265***	(0.235–0.294)

Erreygers-corrected Concentration Indices (CInds) and 95% confidence intervals (CIs) are presented for all outcome variables associated with health care services utilisation. The values presented are parameter estimates of the concentration index (CInd) ranging from -1 (perfect pro-poor inequality) to + 1 (perfect pro-rich inequality). The associated *p*-value helps to determine whether the association also exists in the larger population. Total sample sizes (***N***) for each outcome-year are presented in Table 1. The outcome variable “hospitalisation in the past year” excludes hospital admissions for labour and delivery, including both vaginal birth and Caesarean sections; *p < 0.1; **p < 0.05; ***p < 0.01

*Sources:* PNAD 1998, 2003, and 2008, PNS 2013 and 2019, and the authors’ calculations

The change of CInd values over time reveals somewhat different trends. CInds for preventive screening services decreased over time, indicating a reduction in the utilisation gap between rich and poor. By contrast, although the CInd values increased over time for surgical procedures, no statistically significant differences were identified for physician visits. Furthermore, inequalities with respect to hospital care, as previously depicted in the descriptive charts showing service utilisation by income, indicated a significant pro-poor distribution in 1998 (Erreygers-corrected CInds of -0.004, 95% CI, -0.007–0.001) followed by a reversal to a significant pro-rich inequality in 2019 (Erreygers-corrected CInds of 0.010, 95% CI, 0.004–0.016).

**Fig. 1:**
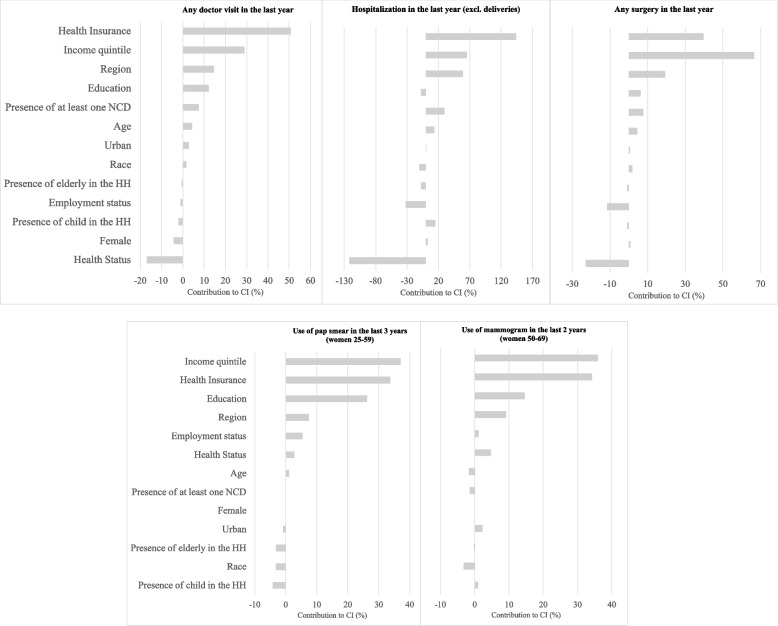
Decomposition of the CInds reveals factors that contribute to inequality in health care utilisation variables (2019). *Sources:* PNAD 1998, 2003, and 2008, PNS 2013 and 2019, and the authors’ calculations. Abbreviations: HH, household; NCD, non–communicable disease

Figure 1 presents the results of the decomposition analysis of the CInds determined from the results of the 2019 survey that document the relative contribution of each critical variable to the overall CInd. The full set of results including all potential variables is included in Additional file 1 Appendix Table A4. The results of the decomposition analysis revealed that the use of private health insurance and socioeconomic status are the two main factors associated with the inequality of health care services utilisation in 2019. Our findings revealed that the use of private health insurance coverage contributed ~ 30%–140%, holding socioeconomic status constant, while differences in socioeconomic status explain ~ 30%–70% of the observed inequalities across all outcomes. Region of residence and educational attainment had positive but relatively smaller contributions to the overall inequality in health care services utilisation. The contributions of all other variables (notably, those identified as predisposing variables) were comparatively small compared to those discussed above.

The inclusion of regional fixed-effects in our analysis of the 2019 wave was intended to capture the contribution of all non-observed differences across the location of residence (e.g., availability of medical equipment, inequality, and/or general standard of living). We performed a series of robustness checks that included some of the non-observed factors that vary at the aggregate level as covariates. The results of this analysis are shown in Additional file 1 Appendix 3 – Robustness tests. In the first test, we substituted regional fixed-effects for state-level covariates that indicate poverty, income inequality, and availability of mammographers, as well as, whether the household was registered at a primary health care facility. The results revealed that state-level poverty, income inequality, and availability of mammographers were positively associated with the pro-rich distribution of utilisation of health care services, whereas being registered in a public health care facility was negatively associated, albeit at a much smaller magnitude. Crucially, the magnitude of the contributions of all other contributing factors does not change significantly. In the second test, we used the Wagstaff correction instead of the Erreygers correction of CInd [36]. The results of this secondary analysis followed the same pattern as our primary findings. Finally, in the third test, we analysed additional preventive care outcomes to determine whether our results represented a more general trend. Among our findings, the decreasing trends we observed with respect to the use of mammograms were also identified in other services that targeted a specific segment of the adult population (i.e., blood sugar tests), but not for other services (i.e., blood pressure and cholesterol testing). These results suggest that the decreasing trends we observed with respect to the use of mammograms, which is a preventive service provided to women only, will also be relevant to services that target the adult population (i.e., blood sugar tests) and also may reflect improvements in primary care services over time [39, 40]. However, the fact that inequalities persist in other preventive services suggests that many of these improvements might not be general in nature.

### Heterogeneity across regions

We used the results of the CInd and decomposition analysis to examine the unequal distribution of health care services utilisation across the five regions of Brazil. The main results of this analysis are shown in Table 3. Our regional-level results for three specific outcomes (i.e., physician visits, use of Pap smears and mammograms) are consistent with findings observed for the entire country. Our analysis of these three outcomes revealed statistically significant inequalities in health care services utilisation that were skewed towards individuals with a higher socioeconomic status in all five regions. By contrast, the magnitude of the CInd representing the utilisation of outpatient and preventive care services was highest in the North-East region of Brazil. However, our consideration of inpatient outcomes (e.g., hospitalisations and surgical procedures) revealed different degrees of inequality across all five regions. With respect to surgical procedures, our analysis revealed a statistically significant pro-rich inequality in all regions, although findings from the South did not achieve statistical significance. Moreover, analysis of these results suggests that the pro-rich skew of hospitalisations in 2019 observed for the entire country was driven by inequalities identified specifically in the North and North-East regions. By contrast, our analysis revealed a statistically significant pro-poor distribution of hospitalisation services in the South region; no statistically significant differences were observed in any of the regions remaining. These results were confirmed by disaggregation of the prevalence of health care services utilisation across income quintiles and regions over time (Additional file 1 Appendix Figures A3 and A4). The decomposition of the CInds calculated on a region-by-region basis using 2019 data (Additional file 1 Appendix Figure A5) confirmed these results. Overall, these findings permitted us to conclude that differences in socioeconomic status and private health insurance coverage result in increased inequalities in the unmet need for health care services that favour the wealthy.

**Table 3:** Erreygers-corrected CInds for health care services utilisation by region

	**South**			**North**			**North-East**			**South-East**				**Midwest**			
**Year**	CInd	95% CI	Obs	CInd	95% CI	Obs	CInd	95% CI	Obs	CInd	95% CI	Obs		CInd	95% CI		Obs
	**Any doctor visit in the past year**																
**1998**	0.110***	(0.091–0.130)	37,788	0.076***	(0.044–0.107)	14,229	0.135***	(0.121–0.149)	66,154	0.100***	(0.087–0.112)	75,583		0.088***	(0.069–0.107)		23,146
**2003**	0.127***	(0.113–0.142)	41,431	0.087***	(0.069–0.106)	25,259	0.144***	(0.132–0.156)	80,087	0.108***	(0.098–0.118)	79,553		0.103***	(0.087–0.119)		27,636
**2008**	0.085***	(0.073–0.097)	41,503	0.067***	(0.046–0.089)	26,020	0.112***	(0.102–0.121)	84,468	0.079***	(0.071–0.088)	83,143		0.084***	(0.069–0.100)		29,904
**2013**	0.104***	(0.071–0.136)	7,548	0.112***	(0.068–0.155)	9,531	0.091***	(0.068–0.114)	18,305	0.118***	(0.093–0.142)	14,293		0.068***	(0.035–0.101)		7,518
**2019**	0.067***	(0.045–0.090)	11,275	0.099***	(0.066–0.132)	12,341	0.118***	(0.101–0.134)	30,699	0.084***	(0.064–0.104)	19,432		0.047***	(0.020–0.074)		10,180
	**Hospitalisation in the past year (excluding labour and delivery)**																
**1998**	-0.012***	(-0.020–-0.004)	37,788	-0.012**	(-0.023–-0.001)	14,229	0.001	(-0.004–0.005)	66,152	-0.000	(-0.005–0.005)	75,585	-0.015***		(-0.023–-0.007)		23,145
**2003**	0.007**	(0.000–0.013)	41,431	0.003	(-0.005–0.011)	25,258	0.012***	(0.007–0.017)	80,083	0.003	(-0.002–0.007)	79,551	-0.008**		(-0.016–-0.001)		27,636
**2008**	-0.006*	(-0.013–0.000)	41,503	-0.011**	(-0.019–-0.003)	26,020	0.006***	(0.002–0.011)	84,468	0.004*	(-0.001–0.008)	83,143	-0.004		(-0.013–0.004)		29,904
**2013**	-0.012	(-0.031–0.006)	7,548	0.009	(-0.015–0.033)	9,531	0.004	(-0.009–0.018)	18,305	0.005	(-0.008–0.017)	14,293	-0.004		(-0.020–0.013)		7,518
**2019**	-0.019***	(-0.033–-0.005)	11,275	0.014**	(0.001–0.026)	12,341	0.021***	(0.012–0.029)	30,699	0.004	(-0.008–0.015)	19,432	0.011		(-0.006–0.028)		10,180
	**Any surgery in the past year**																
**1998**	0.008***	(0.005–0.012)	37,788	0.002	(-0.003–0.008)	14,229	0.008***	(0.005–0.010)	66,152	0.014***	(0.011–0.017)	75,585	0.010***		(0.006–0.015)		23,145
**2003**	0.017***	(0.013–0.021)	41,431	0.005**	(0.000–0.009)	25,258	0.011***	(0.009–0.014)	80,083	0.013***	(0.010–0.016)	79,551	0.011***		(0.007–0.015)		27,636
**2008**	0.010***	(0.006–0.014)	41,503	0.003	(-0.001–0.007)	26,020	0.009***	(0.007–0.011)	84,468	0.013***	(0.010–0.016)	83,143	0.013***		(0.008–0.017)		29,904
**2013**	0.014**	(0.002–0.027)	7,548	0.008	(-0.006–0.022)	9,531	0.013***	(0.005–0.021)	18,305	0.006	(-0.003–0.014)	14,293	0.002		(-0.009–0.014)		7,518
**2019**	0.001	(-0.009–0.010)	11,275	0.012**	(0.002–0.021)	12,341	0.016***	(0.011–0.022)	30,699	0.014***	(0.006–0.022)	19,432	0.013***		(0.004–0.022)		10,180
	**Use of Pap smears in the past three years (women 25–59 years of age)**																
**2003**	0.182***	(0.165–0.198)	14,440	0.198***	(0.160–0.237)	8,570	0.181***	(0.162–0.200)	26,741	0.163***	(0.150–0.177)	27,655	0.182***		(0.158–0.205)	9,733	
**2008**	0.137***	(0.119–0.156)	14,562	0.111***	(0.090–0.132)	9,188	0.126***	(0.108–0.144)	29,191	0.123***	(0.112–0.135)	29,232	0.146***		(0.125–0.167)	10,886	
**2013**	0.168***	(0.118–0.219)	2,793	0.143***	(0.075–0.210)	3,960	0.117***	(0.079–0.155)	7,168	0.146***	(0.107–0.185)	5,449	0.124***		(0.081–0.167)	2,919	
**2019**	0.117***	(0.085–0.149)	3,674	0.081***	(0.038–0.124)	4,502	0.141***	(0.115–0.167)	10,668	0.099***	(0.070–0.127)	6,364	0.151***		(0.109–0.192)	3,570	
	**Use of mammograms in the past two years (women 50–69 years of age)**																
**2003**	0.391***	(0.359–0.424)	4,948	0.314***	(0.263–0.364)	2,109	0.314***	(0.289–0.339)	8,217	0.334***	(0.310–0.357)	9,301	0.404***		(0.362–0.447)		2,614
**2008**	0.298***	(0.269–0.328)	5,619	0.309***	(0.262–0.356)	2,420	0.317***	(0.294–0.340)	9,601	0.255***	(0.233–0.276)	11,078	0.354***		(0.315–0.393)		3,229
**2013**	0.335***	(0.252–0.417)	1,232	0.365***	(0.269–0.460)	1,028	0.275***	(0.213–0.337)	2,594	0.300***	(0.233–0.366)	2,393	0.308***		(0.225–0.391)		1,125
**2019**	0.200***	(0.133–0.266)	2,083	0.257***	(0.182–0.332)	1,870	0.257***	(0.208–0.306)	5,141	0.224***	(0.173–0.274)	3,644	0.150***		(0.080–0.221)		1,780

Erreygers-corrected concentration indices (CInds), 95% confidence intervals (CIs), and total observations for all outcome variables associated with utilisation of health care services are presented; *p < 0.1; **p < 0.05; ***p < 0.01. The values presented are parameter estimates of the concentration index (CInd) ranging from -1 (perfect pro-poor inequality) to + 1 (perfect pro-rich inequality). The associated *p*-value helps to determine whether the association also exists in the larger population

*Sources:* PNAD 1998, 2003, and 2008, PNS 2013 and 2019, and the authors’ calculations

## Discussion

The main objective of this study was to evaluate health care services utilisation in Brazil and identify the main contributors to inequalities over the past 20 years (1998–2019). The data used in this study were collected from two nationally-representative surveys, PNAD conducted in 1998, 2003, and 2008, and PNS conducted in 2013 and 2019, respectively. The results of our analysis revealed a persistent pro-rich inequality with respect to most health care services utilisation outcomes that has diminished somewhat over time. The only exception to this trend is hospitalisations, which moved from an earlier pro-poor inequality to one that is currently pro-rich. Our findings revealed that while the wealthiest individuals were more likely to use preventive, outpatient, and inpatient care services compared to members of the lower socioeconomic groups, there was a consistent downward trend in pro-rich inequalities specifically with respect to the use of preventive health care services. In fact, the use of mammograms and Pap smears, which were the services with the largest initial level of inequality in utilisation were those that showed the largest decline, with both CInds falling over 30% during this period. The results from the decomposition analysis revealed that enabling indicators (e. g., socioeconomic status and private health insurance coverage) are the factors most significantly associated with the observed inequalities. Furthermore, our results are consistent with one another at both national and regional levels. Specifically, we found that the magnitude of inequality with respect to health care services utilisation was particularly large in the North and North-East regions of the country. Socioeconomic factors and private health insurance coverage were also identified as the largest contributors to inequality in these regions. Overall, while our analysis did not permit us to identify the factors driving this downwards trend along with persistent socioeconomic inequalities in health care utilisation, one hypothesis is that they may be related to the large expansion of public policies that granted access to primary health care to a large portion of the population specifically within a context of persistently high income inequality. For example, in 1998 just 5.6% of the population was covered by the FHS, which is Brazil’s main public primary health care program; by 2019, this percentage had grown to 62.7% [41]. Whereas income inequality has decreased between the two time points, Brazil has consistently ranked among the ten countries with the highest level of income inequality worldwide, as measured by the Gini Index [7].

Overall, our results are consistent with those reported in studies from other Latin American countries that have quantified income-related inequalities in health care utilisation [42–48]. Overall, these reports have identified a consistent pro-rich inequality with respect to the utilisation of physician visits and preventive care services, specifically Pap smears and mammograms, throughout Latin America. By contrast, inequalities in the use of hospital care are mixed. Similar to our findings, utilisation of hospital-based health care skews pro-rich in both Mexico [42] and Peru [45]. By contrast, the utilisation of these services is skewed toward the poor in Chile [44]. There was no evidence of inequality in Colombia in a study that specifically targeted older members of the population [46]. In addition, although our findings are consistent with previous results reported for Brazil [20, 23–27], we have observed several new trends during the past decade. Specifically, our results revealed that the earlier pro-poor inequalities in hospitalisations converted to pro-rich over time when we excluded deliveries. Moreover, we found that the pro-rich inequality in utilisation of physician visits and surgical procedures remained unchanged from 2013 through 2019, based on data that were not evaluated in previous publications. Finally, our results that focused on the utilisation of preventive care services, specifically Pap smear and mammogram screening, revealed a continuous trend of declining inequalities, although these remained at higher levels compared to the other health care services. Collectively, these results suggest that there are still significant challenges to be met to provide equitable access to health care in low- and middle-income countries and that these problems persist even after the establishment of a large publicly-funded national health system that provides universal health care coverage to all.

Our finding of pro-rich inequalities in hospitalisation services (excluding those associated with labour and delivery) in 2019 complements the results reported in the existing literature. By covering the entire adult population (instead of just the elderly population [27]) and excluding deliveries from total hospitalisations, our results provide a clearer understanding of the direction of inequality with respect to the need-based utilisation of inpatient care services. These findings indicate that the rate of utilisation for those 18 years of age or older in the lowest income quintile decreased significantly across most areas of Brazil, particularly in the comparatively impoverished North and Northeast regions. Furthermore, decomposition analysis of the CInds for hospitalisation services suggested that the observed pro-rich inequality may be largely driven by the availability of private insurance to individuals in the higher income groups.

Another important finding relates to the downward trend in the pro-rich inequality observed in the utilisation of preventative health care services (i.e., mammograms and Pap smear) between 2003 and 2019. This result is particularly relevant because it coincides with a period during which both the coverage and quality of primary health care services increased in Brazil [9, 10]. Moreover, when we include a covariate in the model indicating coverage of the household by the FHS, the results of the decomposition of CInd for the preventive care services, specifically Pap smears, revealed that being covered by this service was negatively associated with the pro-rich inequality in utilisation of the health care services. This result indicates that the program has effectively targeted the poorest groups (see Additional file 1 Appendix 3 – Robustness tests). This result also complements findings from previous studies that have included these recent findings (up to 2019) [25–27]. Although these findings suggest that the gap between the rich and the poor with respect to the use of preventive health care services has been closing, there remains significant variation across the regions in the country. Persistent geographic disparities in the use of mammograms could be explained at least in part by the unequal distribution of imaging equipment. Amaral et al. [49] reported that while overall mammography capacity is sufficient to meet existing needs in Brazil, specific resources are not distributed proportionally based on the population of each region. Notably, only the South region has mammography equipment that is sufficient to serve the entire population.

As noted above, our findings documenting health care services utilisation at the regional level are consistent with those that have emerged at the national level. Both analyses point to significant inequalities, specifically in the North-East, when compared to the rest of the country. This is most likely a result of both economic and healthcare-related factors. When ranked by region, the North-East has one of the lowest levels of socioeconomic development. The North-East is currently ranked last of the five designated regions in Brazil with respect to Gross Regional Product per capita [50]. The high concentration of inequalities in the North-East may also reflect the limited availability of health care infrastructure. Of note, our findings suggested a large improvement in utilisation of preventive care services among poorer women across all regions of Brazil, with the largest improvements reported for the Midwest region and revealing a clear trend of improvement over time across all regions [51, 52].

When considering barriers to health care, the results of our study are comparable to those reported previously. Collectively, our findings suggested that the availability of private health care insurance is positively associated with the pro-rich inequalities in health care services utilisation in Brazil. The use of private health insurance is tied directly to employment status and the ability to pay for care and is thus concentrated in regions with the highest income levels. The availability of private insurance tends to exacerbate inequalities in health care services utilisation and provides users with advantages over those who rely solely on public health services [53]. This may lead to an implicit two-tier system, in which those with higher income have ‘double coverage’ from both private health insurance and the national system. While two-tier systems exist in other Latin American countries [54], Brazil’s case is unique given that an extensive private system co-exists with a national health service that provides health care coverage for all. Private medical insurance spending in Brazil is the highest in the region; this is coupled with public spending on healthcare which is well below the average for Latin American nations [7]. Focusing specifically on Brazil, where there is a large body of evidence indicating the existence of ongoing socioeconomic inequalities in many dimensions of health (i.e., inequalities in health according to socioeconomic status), our results suggest that lower rates of utilisation of preventive health care services might be one of the reasons why lower-income individuals present, on average, with inferior health outcomes.

This study has some limitations. While Andersen’s behavioral model of health services utilisation [30] provides a framework that permits us to consider many of these factors and their interrelationships, it exhibits only limited capacity to generalise these results. The Andersen model also offers guidance toward an appropriate interpretation of these results by facilitating the identification of the factors that contribute to income-associated inequalities in health care services utilisation. Likewise, as applied to our results, the Cind and decomposition analysis facilitate the acquisition of correlational data and do not provide insight into causal inference. Furthermore, socioeconomic inequalities measured by the Cind (i.e., based on income distribution) represent only a partial approach to health care inequalities. While the focus of our analysis is on income-related inequalities, other potentially relevant factors, such as racial and gender-based inequalities have not been considered. For example, results from a previous study revealed that multiple inequities play a much larger role than income-related inequity alone [55]. There are some further limitations associated with the decomposition analysis used in this paper as previously reported [56]. First, it is one-dimensional because it focuses on the degree of variation in health but ignores rank; second, it can only correctly decompose one form of rank dependent index because it assumes a constant weighting function [56]. Finally, as discussed in the text, we needed to exclude individuals residing in the rural areas of the North region to maintain sample consistency over time. This may have led to an underestimate of inequalities, as access to health care services is particularly restricted in this part of the country [57]. Nonetheless, and despite these limitations, we present a full overview and analysis of the inequalities in health care services utilisation in Brazil over two decades. Our study has revealed the main factors that contribute to these inequalities and identified private health insurance as a key driver of these observations in Brazil.

## Conclusion

Our results support the view that efforts to achieve equality in the delivery of health care services need to be intensified and should rank high among policy concerns in Brazil. Our findings also have implications for policy development in other middle-income countries that provide universal health care coverage. While the Brazilian system has a unique public/private health care mix, our evaluation still offers valuable lessons for other middle-income countries in Latin America and beyond. Specifically, our results reveal that reductions in inequalities in access to health care can be achieved in a system in which public and private financing systems co-exist. However, caution should be exercised as some of the challenges associated with a dual (public/private) system tend to be persistent. First, our findings are consistent with results reported by several Organisation for Economic Cooperation and Development (OECD)-aligned countries and the Americas that have achieved universal or near-universal health care coverage for their populations. Specifically, OECD findings revealed that inequalities in access to health care services persist over time despite increases in health care expenditures and investments directed at expanding critical infrastructure [2, 3, 58, 59]. Second, our findings revealed that, although utilisation of health care services has increased over time, inequalities in health care services utilisation remain high and favour those of higher socioeconomic status; this is particularly the case for the utilisation of preventive care services. While the reduction in inequalities observed may have been the direct result of programmes and policies implemented over the last few years (e.g., FHS), this question goes beyond the scope of this paper. More importantly, the first decade of the 2000s coincided with significant economic growth. This may have directly or indirectly resulted in an overall reduction in the pro-rich inequality with respect to access to the selected interventions. Policy interventions might be developed to target both financial and non-financial barriers, particularly those with the greatest impact on poor and vulnerable individuals residing in the lower-resourced regions of the country. Moreover, given the apparent associations between these inequalities and the availability of private health insurance, future research might be designed to explore the possibility of a causal relationship between the availability of this type of health care coverage and the utilisation of health services.

## Supplementary Information

**Additional file 1.**

## Data Availability

Data are available upon request.

## Abbreviations

Cinds: Concentration Indices
IBGE: Instituto Brasileiro de Geografia e Estatística
OECD: Organisation for Economic Cooperation and Development
PNAD: Pesquisa Nacional por Amostra de Domicílios
PNS: Pesquisa Nacional de Saúde
SUS: Sistema Único de Saude

## References

[1] United Nations. Transforming our world: the 2030 agenda for sustainable developmen^t.^ 70th Session of the United Nations General Assembly, 21 October 2015; New York: UN; 2019. Available from: http://www.un.org/en/ga/search/view_doc.asp?symbol=A/ RES/70/1 Accessed 20 April 2022.

[2] Organization for Economic Cooperation and Development. Unmet needs for health care: comparing approaches and results from international surveys. Paris: OECD; 2020. Available from https://www.oecd.org/health/health-systems/Unmet-Needs-for-Health-Care-Brief-2020.pdf. Accessed 20 June 2022.

[3] Devaux M (2015). Income-related inequalities and inequities in health care services utilisationation in 18 selected OECD countries. Eur J Health Econ.

[4] Cite Mossialos, E, Dixon, A. Funding health care in Europe: weighing up the options. In: Mossialos E, Dixon A, Figueras J, Kutzin J, editors. Funding Health Care: Options for Europe. Buckingham. London: Open University Press; 2002.

[5] Cookson R, Propper C, Asaria M, Raine R (2016). Socio-economic inequalities in health Care in England. Fisc Stud.

[6] Xu Y, Zhang T, Wang D (2019). Changes in inequality in utilization of preventive care services: evidence on China’s 2009 and 2015 health system reform. Int J Equity Health.

[7] The World Bank, World Development Indicators (2020). Gini index, Brazil . Retrieved from https://data.worldbank.org/indicator/SI.POV.GINI?locations=BR.

[8] Rocha R, Furtado I, Spinola P (2021). Financing needs, spending projection, and the future of health in Brazil. Health Econ.

[9] Mrejen M, Rocha R, Millett C, Hone T. (2021). The quality of alternative models of primary health care and morbidity and mortality in Brazil: a national longitudinal analysis. The Lancet Regional Health - Americas, 4, 100034. 10.1016/j.lana.2021.100034. 10.1016/j.lana.2021.100034 PMC9903814 36776706

[10] Hone T, Saraceni V, Medina Coeli C, Trajman A, Rasella D, Millett C, Durovni B (2020). Primary healthcare expansion and mortality in Brazil’s urban poor: a cohort analysis of 1.2 million adults. PLoS medicine.

[11] Rocha R, Soares RR (2010). Evaluating the impact of community-based health interventions: evidence from Brazil’s family health program. Health Econ.

[12] Hone T, Rasella D, Barreto ML, Majeed A, Millett C (2017). Association between expansion of primary healthcare and racial inequalities in mortality amenable to primary care in Brazil: a national longitudinal analysis. PLoS Med.

[13] Castro MC, Massuda A, Almeida G, Menezes-Filho NA, Andrade MV, de Souza Noronha KVM, Rocha R, Macinko J, Hone T, Tasca R (2019). Brazil’s unified health system: the first 30 years and prospects for the future. Lancet.

[14] IBGE (2020). Pesquisa nacional de saúde, 2019: Informações sobre domicílio, acesso e utilização dos serviços de saúde. Instituto Brasileiro de Geografia e Estatística – IBGE.

[15] Bossert T, Blanchet N, Sheetz S, Pinto D, Cali J, Perez Cuevas R (2014). Comparative review of health system integration in selected countries in Latin America.

[16] Miranda VIA, Schafer AA, Tomasi CD, Soratto J, de Oliveira Meller F, Silveira MPT (2021). Inequalities in access to medicines for diabetes and hypertension across the capitals in different regions of Brazil: a population-based study. BMC Public Health.

[17] França GV, Restrepo-Mendez MC, Maia MFS, Victora CG, Barros AJ (2016). Coverage and equity in reproductive and maternal health interventions in Brazil: impressive progress following the implementation of the unified health system. Int J Equity Health.

[18] Facchini LA, Piccini RX, Tomasi E, Thum E, Teixeira VA, Silveira DS, Maia MD, Siqueira FV, Rodrigues MA, Paniz VV (2008). Evaluation of the effectiveness of primary health care in south and northeast Brazil: methodological contributions. Cad Saúde Pública.

[19] Pinto LF, Giovanella L (2018). The family health strategy: expanding access and reducing hospitalizations due to ambulatory care sensitive conditions (acsc). Cien Saude Colet.

[20] Andrade MV, Noronha KV, Menezes RD, Souza MN, Reis CD, Martins DR, Gomes L (2013). Desigualdade socioeconômica no acesso aos serviços de saúde no brasil: um estudo comparativo entre as regiões brasileiras em 1998 e 2008. Econ Aplicada.

[21] Garnelo L, Parente RCP, Puchiarelli MLR, Correia PC, Torres MV, Herkrath FJ (2020). Barriers to access and organization of primary health care services for rural riverside populations in the Amazon. Int J Equity Health.

[22] Garcia-Subirats I, Vargas I, Mogollón-Pérez AS, de Paepe P, da Silva MRF, Unger JP, Vázquez ML (2014). Barriers in access to healthcare in countries with different health systems. A cross-sectional study in municipalities of central Colombia and north-eastern Brazil. Soc Sci Med.

[23] Macinko J, Lima-Costa MF (2012). Horizontal equity in health care utilization in Brazil, 1998–2008. Int J Equity Health.

[24] Almeida G, Sarti FM, Ferreira FF, Diaz MDM, Campino ACC (2013). Analysis of the evolution and determinants of income-related inequalities in the Brazilian health system, 1998–2008. Rev Panam Salud Publica.

[25] Politi R (2014). Desigualdade na utilização de serviços de saúde entre adultos: uma análise dos fatores de concentração da demanda. Economia Aplicada.

[26] Mullachery P, Macinko J, Silver D (2020). Have Health Reforms in Brazil Reduced Inequities in Access to Cancer Screenings for Women?. J Ambulatory Care Manage.

[27] Dos Santos AMA, Triaca LM, Tejada CAO (2021). Evolution of inequalities in health care use among older people in Brazil: Evidence for the period 1998–2019. J Econ Ageing.

[28] IBGE (2020). Um panorama da saúde no Brasil: acesso e utilização dos serviços, condições de saúde e fatores de risco e proteção à saúde 2008. Instituto Brasileiro de Geografia e Estatística – IBGE.

[29] IBGE (2014). Pesquisa nacional de saúde, 2013: percepção do estado de saúde, estilos de vida e doenças crônicas. Brasil, grandes regiões e unidades da federação. Instituto Brasileiro de Geografia e Estatística – IBGE.

[30] Andersen RM (1995). Revisiting the behavioral model and access to medical care: does it matter?. J Health Soc Behav Pages.

[31] Brasil. Diretrizes brasileiras para o rastreament do cancer do colo do utero. Rio de Janeiro, Brazil: Instituto Nacional de Cancer (INCA); 2011. Retrieved from http://bvsms.saude.gov.br/bvs/publicacoes/inca/rastreamento cancer colo utero.pdf

[32] Brasil. Diretrizes para a deteccao precoce do Cancer de Mama no Brasil. Rio de Janeiro, Brazil: Instituto Nacional de Cancer (INCA); 2014. Retrieved from http://www.saude.pr.gov.br/arquivos/File/Deteccao_precoce_CANCER_MAMA_INCA.pdf

[33] Van Doorslaer E, Van Ourti T. Measuring inequality and inequity in health and health care. In: Glied S, Smith PC, editors. The Oxford handbook of health economics. London; 2012.

[34] Erreygers G (2009). Correcting the concentration index. J Health Econ.

[35] Kjellsson G, Gerdtham U-G (2013). On correcting the concentration index for binary variables. J Health Econ.

[36] Ataguba JE (2022). A short note revisiting the concentration index: Does the normalization of the concentration index matter?. Health Econ.

[37] Wagstaff A (2002). Inequality Aversion, Health Inequalities and Health Achievement. J Health Econ.

[38] Van Doorslaer E, Koolman X, Jones AM (2004). Explaining income-related inequalities in doctor utilisation in Europe. Health Econ.

[39] Clarke L, Anderson M, Anderson R, Klausen MB, Forman R, Kerns J, Rabe A, Kristensen SR, Theodorakis P, Valderas J, Kluge H, Mossialos E (2021). Economic Aspects of Delivering Primary Care Services: An Evidence Synthesis to Inform Policy and Research Priorities. Milbank Q.

[40] Masseria C, Irwin R, Thomson S, Gemmill M, Mossialos E (2009). Primary Care in Europe.

[41] Rocha R, Mrejen M. e M. Coube (2020). Um Decreto para Estradas e a Estrada da Saúde no Brasil. Nota Técnica n.13. IEPS: São Paulo.

[42] Barraza-Lloréns M, Panopoulou G, D.az, B. Y.  (2013). Income-related inequalities and inequities in health and health care utilization in Mexico, 2000–2006. Rev Panam Salud Publica.

[43] Ruiz Gómez F, Zapata Jaramillo T, Garavito Beltrán L (2013). Colombian health care system: results on equity for five health dimensions, 2003–2008. Rev Panam Salud Publica.

[44] Vasquez F (2013). Income-related inequality in health and health care utilization in Chile, 2000–2009. Rev Panam Salud Pública.

[45] Petrera M, Valdivia M, Jimenez E, Almeida G (2013). Equity in health and health care in Peru, 2004–2008. Rev Panam Salud Publica.

[46] Garcia-Ramirez J (2020). Inequality in healthcare use among older people in Colombia. Int J Equity Health.

[47] Palacios A, Espinola N, Rojas-Roque C (2020). Need and inequality in the use of health care services in a fragmented and decentralized health system: evidence for Argentina. Int J Equity Health.

[48] González C, Triunfo P (2020). Horizontal inequity in the use and access to health care in Uruguay. Int J Equity Health.

[49] Amaral P, Luz L, Cardoso F, Freitas R (2017). Spatial distribution of mammography equipment in Brazil. Rev Brasileira de Estudos Urbanos e Regionais (RBEUR).

[50] IBGE (2019). Sistema de Contas Regionais: Brasil 2019. Retrieved from: https://biblioteca.ibge.gov.br/visualizacao/livros/liv101873_informativo.pdf.

[51] De La Torre A, Nikoloski Z, Mossialos E (2018). Equity of access to maternal health interventions in Brazil and Colombia: a retrospective study. Int J Equity Health.

[52] Peroni F, d. M. A., Lindelow, M., De Souza, D. O., and Sjoblom, M.  (2019). Realizing the right to health in Brazil’s unified health system through the lens of breast and cervical cancer. Int J Equity Health.

[53] Béhague DP, Goncalves H, Dias Da Costa J (2002). Making medicine for the poor: primary health care interpretations in Pelotas, Brazil. Health Policy Planning.

[54] Sapelli C (2004). Risk segmentation and equity in the Chilean mandatory health insurance system. Soc Sci Med.

[55] Barbosa EC, Cookson R (2019). Multiple inequity in health care: an example from Brazil. Soc Sci Med.

[56] Heckley G, Gerdtham UG, Kjellsson G (2016). A general method for decomposing the causes of socioeconomic inequality in health. J Health Econ.

[57] Rocha, R.; Camargo, M. ; Falcao, L. ; Silveira, M. ; Thomazinho, G. . A (2021). Saúde na_Amazônia Legal: Evolução Recente e Desafios em Perspectiva Comparada. Health in the Legal Amazon: Recent Evolution and Challenges in Comparative Perspective. Retrieved from: https://amazonia2030.org.br/wp-content/uploads/2021/11/A-Saude-na-Amazonia-Legal.pdf

[58] Báscolo, E., Houghton, N., & Del Riego, A. (2020). Leveraging household survey data to measure barriers to health services access in the Americas. Revista Panamericana de Salud Pública. 10.26633/RPSP.2020.100 PMC7429927 32821260

[59] Coube M, Zlatko N, Matias M, Elias M. “Inequalities in unmet need for health care services and medications in Brazil: a decomposition analysis”. The Lancet Regional Health - Americas. 2023;19:100426. 10.1016/j.lana.2022.100426 PMC10025415 36950032

