# Supplementary material for: Persistent inequalities in health care services utilisation in Brazil (1998–2019)
Source: Int J Equity Health. 2023 Feb 2;22:25. doi: 10.1186/s12939-023-01828-3 (PMC9893569; doi:10.1186/s12939-023-01828-3)
Supplement: Supplementary file 1 — Additional file 1. [file 12939_2023_1828_MOESM1_ESM.docx]

***Supplementary materials***

[*Appendix 1. The procedure used to identify the main dependent variables* 2](#_Toc115346841)

[*Appendix 2. Full description of variables used in the modelling exercise* 3](#_Toc115346842)

[*Appendix Tables* 5](#_Toc115346843)

[*Appendix 3. Robustness tests* 22](#_Toc115346844)

# *Appendix 1. The procedure used to identify the main dependent variables*

Measures of health care services utilization were based on responses to the following questions, with outcomes shown in parentheses:

- “In the last 12 months, the person consulted with a doctor?” (*Any doctor visit in the past year*);
- “The person was hospitalized for at least 24 hours during the last year?” (*Hospitalization in the past year, excluding labour and delivery*);
- “What was the main service received when hospitalized during the last 12 months?” (Options: surgery, psychiatric care, clinical care, vaginal birth, C-section, complementary diagnostic exams, other) (*Any surgery in the past year*);
- “When was the last time you had cervical cancer screening?” (*Use of Pap smears in the past three years by women between the ages of 25 and 59 years*) and
- “When was the last time you had a mammogram?” (*Use of mammograms in the past two years by women between the ages of 50 and 69 years*).

Note that in the outcome listed as hospitalisations in the past year, we have excluded admissions for labour and delivery for both vaginal births and Caesarean sections. These respondents were coded as zero, i.e., as having had no hospital admissions during the past year. In-hospital deliveries represented 24.4% and 11.9% of total hospitalisations in Brazil in 1998 and 2019, respectively.

The outcome variable ‘Any surgery in the past year’ is coded 1 if the respondent answered ‘yes’ to the option ‘surgery’ when asked about the main service received when hospitalised during the past 12 months. This variable was coded 0 if the respondent has not been hospitalised or had been hospitalised for an indication other than surgery (i.e., psychiatric care, clinical care, vaginal birth, C-section, complementary diagnostic exams, other).

The two preventive care outcome variables (Pap smears and mammograms) were calculated by restricting the sample to eligible women only using age ranges and screening intervals based on guidelines from the Brazilian Ministry of Health.^[1-2]^ Questions regarding the use of preventive health care services were included in surveys beginning in 2003.

**References:**

1. Brasil. (2011). Diretrizes brasileiras para o rastreament do cancer do colo do utero. Rio de Janeiro, Brazil: Instituto Nacional de Cancer (INCA). Retrieved from [http://bvsms.saude.gov.br/bvs/publicacoes/inca/rastreamento cancer colo utero.pdf](http://bvsms.saude.gov.br/bvs/publicacoes/inca/rastreamento%20cancer%20colo%20utero.pdf)
2. Brasil. (2014). Diretrizes para a deteccao precoce do Cancer de Mama no Brasil. Rio de Janeiro, Brazil: Instituto Nacional de Cancer (INCA). Retrieved from <http://www.saude.pr.gov.br/arquivos/File/Deteccao_precoce_CANCER_MAMA_INCA.pdf>

# *Appendix 2. Full description of variables used in the modelling exercise*

To model the determinants of health care services utilisation, we followed Anderson’s behavioral model and grouped the covariates into three blocks of contributing factors for the decomposition analysis, i.e., predisposing factors, enabling factors, and need factors. Predisposing factors include individual characteristics related to demographic and social structure (e.g., employment, social class, occupation, race). Enabling factors are those that involve the resources specific to individuals which may have an impact on their access to the services they need (e.g., income, health insurance) and the resources available in the community in which they live (e.g., physicians and availability of hospital beds). Finally, the term need factors refers to the need for medical care, either individually perceived or assessed by providers. This model assumes that, in an equitable system, need factors should be the primary reason for any observed variations in the use of health care services. It also distinguishes factors that might be changed more readily by the health care system (i.e., enabling factors) from those that are not (predisposing factors).

Our analysis of predisposing factors included gender (indicating whether the individual is female), age categorized into groups (18–24 years, 25–34 years, 35–44 years, 45–54 years, 55–64 years, 65 years or greater), race (white, Black, Asian, mixed/brown or native), educational attainment (none, incomplete elementary, complete elementary school, incomplete secondary, complete secondary, incomplete higher or complete higher), employment status (inactive, unemployed, or employed) and a binary variable indicating presence or absence of children under 5 years of age or elderly individuals (60 years of age and older) living in the household. Enabling factors included income quintile (calculated based on household monthly income per capita at constant prices of 2019 in Brazilian currency)^^[[1]](#footnote-1)^^, a binary variable indicating the location of a household in an urban area and region of residence (North, North–East, Midwest, South–East or South)^^[[2]](#footnote-2)^^, and a binary variable indicating whether or not the individual holds any private health insurance. Finally, two covariates related to health needs were addressed, including a binary variable indicating a self-reported diagnosis of one or more non–communicable diseases (including chronic back pain, arthritis or rheumatism, cancer, diabetes, hypertension, heart disease, chronic kidney disease, or depression)^^[[3]](#footnote-3)^^ and health status (very good, good, regular, poor, very poor).

An analysis of the summary statistics is depicted in Appendix Table A1. From this analysis, we observe that the Brazilian adult population is becoming older and more educated. Our findings also reveal that most of the adults live in the wealthiest regions of the country (South, Southeast, and Northeast). In addition, while the rate of unemployment was declining between 1998 and 2013, it has since increased, most likely as a result of the 2014–2016 recession in Brazil. Similarly, despite a large increase in the prevalence of private health insurance between 1998 and 2013, our findings revealed no significant changes in the share of respondents reporting that they were covered by private health insurance in the past six years. Our results also point to several significant changes in the variables related to the self-reported prevalence of non-communicable diseases and health status. The proportion of respondents who reported at least one chronic disease increased by 6.5 percentage points during the period analyzed.

**References:**

1. Wagstaff, Adam & van Doorslaer, Eddy & Watanabe, Naoko, 2003. "On decomposing the causes of health sector inequalities with an application to malnutrition inequalities in Vietnam," Journal of Econometrics, Elsevier, vol. 112(1), pages 207-223, January.

# *Appendix Tables*

Table A1. Summary statistics for hospitalization variables.

|  | **1998** (***N*** = 215,483) | | |  | **2003** (***N*** = 252,108) | | |  | **2008** (***N*** = 263,902) | | |  | **2013** (***N*** = 57,195) | | |  | **2019** (***N*** = 83,927) | | |
| --- | --- | --- | --- | --- | --- | --- | --- | --- | --- | --- | --- | --- | --- | --- | --- | --- | --- | --- | --- |
| **Variable** | n | % | 95% CI |  | n | % | 95% CI |  | n | % | 95% CI |  | n | % | 95% CI |  | n | % | 95% CI |
| **Hospitalization in the last year** |  |  |  |  |  |  |  |  |  |  |  |  |  |  |  |  |  |  |  |
| - All admissions | 18,316 | 8.5 | (8.3–8.7) |  | 20,421 | 8.1 | (8–8.3) |  | 21,376 | 8.1 | (7.9–8.2) |  | 4,004 | 7.0 | (6.7–7.4) |  | 6,378 | 7.6 | (7.2–7.9) |
| - Excluding deliveries | 14,006 | 6.5 | (6.3–6.6) |  | 16,387 | 6.5 | (6.4–6.7) |  | 17,681 | 6.7 | (6.6–6.9) |  | 3,432 | 6.0 | (5.7–6.3) |  | 5,455 | 6.5 | (6.2–6.9) |
| - Deliveries only | 4,525 | 2.1 | (2–2.1) |  | 4,034 | 1.6 | (1.5–1.6) |  | 3,431 | 1.3 | (1.3–1.4) |  | 629 | 1.1 | (0.9–1.2) |  | 923 | 1.1 | (1–1.2) |

*Notes*: The findings presented include the mean prevalence for all outcome variables in percentage (%) and the number of observations (n) and 95% confidence intervals (CIs). ***N*** refers to the total sample of the adult population (18 years and older) which corresponds to the total sample size of each survey year.

*Sources:* PNAD 1998, 2003, and 2008, PNS 2013 and 2019, and the authors’ calculations.

Table A2. Summary statistics of the independent variables

|  | **1998** (***N*** = 215,470) | |  | **2003** (***N*** = 252,108) | |  | **2008** (***N*** = 263,902) | |  | **2013** (***N*** = 57,195) | |  | **2019** (***N*** = 83,927) | |
| --- | --- | --- | --- | --- | --- | --- | --- | --- | --- | --- | --- | --- | --- | --- |
|  | % | 95% CI |  | % | 95% CI |  | % | 95% CI |  | % | 95% CI |  | % | 95% CI |
| **PREDISPOSING FACTORS** | | |  |  |  |  |  |  |  |  |  |  |  |  |
| *Female* | 52.1 | (51.9–52.2) |  | 52.2 | (52.1–52.4) |  | 52.4 | (52.2–52.6) |  | 52.9 | (52.9–52.9) |  | 53.2 | (53.2–53.2) |
| *Age* |  |  |  |  |  |  |  |  |  |  |  |  |  |  |
| 18-24 | 20.3 | (20–20.5) |  | 20.2 | (20–20.4) |  | 17.5 | (17.4–17.7) |  | 15.9 | (15.9–15.9) |  | 13.9 | (13.9–13.9) |
| 25-34 | 24.3 | (24–24.6) |  | 23.5 | (23.3–23.7) |  | 23.2 | (23–23.5) |  | 21.7 | (21.3–22) |  | 18.1 | (17.8–18.4) |
| 35-44 | 21.2 | (21–21.5) |  | 20.7 | (20.5–21) |  | 20.3 | (20.1–20.5) |  | 19.1 | (18.6–19.6) |  | 20.2 | (19.8–20.7) |
| 45-54 | 14.8 | (14.6–15) |  | 15.6 | (15.4–15.8) |  | 16.8 | (16.7–17) |  | 17.5 | (17.1–17.9) |  | 17.9 | (17.5–18.2) |
| 55-64 | 9.8 | (9.6–10) |  | 10 | (9.8–10.1) |  | 11.1 | (10.9–11.2) |  | 13.4 | (13–13.9) |  | 15 | (14.7–15.4) |
| 65 or older | 9.6 | (9.4–9.8) |  | 10 | (9.8–10.1) |  | 11 | (10.8–11.2) |  | 12.3 | (12–12.6) |  | 14.9 | (14.7–15.1) |
| *Race* |  |  |  |  |  |  |  |  |  |  |  |  |  |  |
| White | 55.1 | (54.4–55.9) |  | 52.8 | (52.3–53.4) |  | 49.7 | (49.2–50.2) |  | 47.7 | (46.9–48.5) |  | 43.3 | (42.6–44) |
| Black | 6.1 | (5.8–6.3) |  | 6.4 | (6.2–6.6) |  | 7.6 | (7.3–7.8) |  | 9.2 | (8.7–9.6) |  | 11.5 | (11.1–11.9) |
| Asian | 0.6 | (0.5–0.7) |  | 0.5 | (0.4–0.6) |  | 0.7 | (0.6–0.7) |  | 0.9 | (0.8–1.1) |  | 0.9 | (0.8–1) |
| Browns/Mixed | 37.9 | (37.2–38.7) |  | 40.1 | (39.6–40.6) |  | 41.7 | (41.3–42.2) |  | 41.8 | (41–42.5) |  | 43.7 | (43–44.4) |
| Indigenous | 0.2 | (0.2–0.3) |  | 0.2 | (0.2–0.2) |  | 0.3 | (0.3–0.3) |  | 0.4 | (0.3–0.5) |  | 0.5 | (0.4–0.6) |
| *Education* |  |  |  |  |  |  |  |  |  |  |  |  |  |  |
| None | 17.2 | (16.7–17.6) |  | 14.1 | (13.7–14.4) |  | 11.9 | (11.6–12.2) |  | 13.6 | (13.1–14.2) |  | 6 | (5.8–6.3) |
| Basic incomplete | 46.4 | (45.8–46.9) |  | 40.4 | (40–40.9) |  | 33.9 | (33.5–34.2) |  | 25 | (24.3–25.8) |  | 28.3 | (27.7–28.9) |
| Basic complete | 8.8 | (8.6–8.9) |  | 9.1 | (9–9.3) |  | 9.7 | (9.6–9.9) |  | 9.9 | (9.5–10.4) |  | 7.8 | (7.5–8.1) |
| Secondary incomplete | 5.5 | (5.3–5.6) |  | 6.2 | (6–6.3) |  | 6.2 | (6.1–6.3) |  | 5.5 | (5.2–5.9) |  | 6.8 | (6.4–7.1) |
| Secondary complete | 14.2 | (13.9–14.5) |  | 19.9 | (19.6–20.2) |  | 24.9 | (24.6–25.2) |  | 28.3 | (27.7–29) |  | 30 | (29.4–30.5) |
| Higher incomplete | 2.6 | (2.5–2.7) |  | 3.8 | (3.7–3.9) |  | 4.7 | (4.5–4.8) |  | 4.7 | (4.4–5) |  | 5.2 | (4.9–5.5) |
| Higher complete | 5.4 | (5.1–5.6) |  | 6.5 | (6.2–6.7) |  | 8.7 | (8.4–9) |  | 12.8 | (12.1–13.5) |  | 16 | (15.3–16.6) |
| *Employment status* |  |  |  |  |  |  |  |  |  |  |  |  |  |  |
| Inactive | 30.8 | (30.4–31.1) |  | 29.7 | (29.3–30) |  | 29.1 | (28.8–29.4) |  | 35 | (34.4–35.6) |  | 33.4 | (32.9–33.9) |
| Unemployed | 5.5 | (5.3–5.6) |  | 6.3 | (6.1–6.4) |  | 4.6 | (4.5–4.7) |  | 3.4 | (3.2–3.7) |  | 5.3 | (5–5.6) |
| Employed | 63.8 | (63.4–64.1) |  | 64.1 | (63.7–64.4) |  | 66.3 | (66–66.6) |  | 61.6 | (60.9–62.2) |  | 61.3 | (60.7–61.8) |
| *Presence of elderly in the HH* | 28.4 | (27.9–28.8) |  | 28.6 | (28.2–29) |  | 30.1 | (29.7–30.5) |  | 31.6 | (31.1–32.2) |  | 34.2 | (33.8–34.6) |
| *Presence of a child in the HH* | 32.1 | (31.6–32.6) |  | 28.4 | (28–28.8) |  | 23.9 | (23.5–24.2) |  | 21.3 | (20.7–21.8) |  | 20 | (19.6–20.5) |
| **ENABLING FACTORS** |  |  |  |  |  |  |  |  |  |  |  |  |  |  |
| *Income quintile* |  |  |  |  |  |  |  |  |  |  |  |  |  |  |
| Poorest quintile | 20.1 | (19.5–20.7) |  | 20.4 | (20–20.9) |  | 20 | (19.6–20.5) |  | 20.1 | (19.5–20.7) |  | 20 | (19.5–20.5) |
| Poor quintile | 20.2 | (19.8–20.7) |  | 19.6 | (19.3–20) |  | 20 | (19.7–20.4) |  | 20 | (19.3–20.6) |  | 20.1 | (19.6–20.7) |
| Average quintile | 19.8 | (19.4–20.2) |  | 20 | (19.7–20.4) |  | 19.9 | (19.6–20.3) |  | 20 | (19.4–20.7) |  | 19.9 | (19.4–20.4) |
| Rich quintile | 19.9 | (19.5–20.3) |  | 19.9 | (19.5–20.2) |  | 20 | (19.7–20.3) |  | 20.1 | (19.4–20.7) |  | 21 | (20.5–21.5) |
| Richest quintile | 20 | (19.3–20.6) |  | 20 | (19.5–20.6) |  | 20 | (19.5–20.5) |  | 19.9 | (19.1–20.7) |  | 19 | (18.3–19.7) |
| *Area of residence* |  |  |  |  |  |  |  |  |  |  |  |  |  |  |
| Rural | 19.2 | (18.1–20.3) |  | 14.9 | (14–15.7) |  | 14.1 | (13.3–15) |  | 12.2 | (11.8–12.6) |  | 12.1 | (11.8–12.5) |
| Urban | 80.8 | (79.7–81.9) |  | 85.1 | (84.3–86) |  | 85.9 | (85–86.7) |  | 87.8 | (87.4–88.2) |  | 87.9 | (87.5–88.2) |
| *Region* |  |  |  |  |  |  |  |  |  |  |  |  |  |  |
| South | 15.3 | (15.3–15.3) |  | 14.8 | (14.8–14.8) |  | 14.7 | (14.7–14.7) |  | 14.8 | (14.8–14.8) |  | 14.7 | (14.7–14.7) |
| North | 5 | (5–5) |  | 5.9 | (5.9–5.9) |  | 6.3 | (6.3–6.3) |  | 7.5 | (7.5–7.5) |  | 7.8 | (7.8–7.8) |
| North-East | 29 | (29–29) |  | 28.7 | (28.7–28.7) |  | 28.5 | (28.5–28.5) |  | 26.5 | (26.5–26.5) |  | 26.5 | (26.5–26.5) |
| South-East | 43.7 | (43.7–43.7) |  | 43.4 | (43.4–43.4) |  | 43.1 | (43.1–43.1) |  | 43.9 | (43.9–43.9) |  | 43.4 | (43.4–43.4) |
| Midwest | 7 | (7–7) |  | 7.1 | (7.1–7.1) |  | 7.4 | (7.4–7.4) |  | 7.4 | (7.4–7.4) |  | 7.6 | (7.6–7.6) |
| *Private Health Insurance* | 25.8 | (25.2–26.5) |  | 26.3 | (25.7–26.8) |  | 28 | (27.5–28.5) |  | 30.3 | (29.4–31.2) |  | 29.8 | (29–30.6) |
| **NEED FACTORS** |  |  |  |  |  |  |  |  |  |  |  |  |  |  |
| *Presence of at least one NCD* | 43.8 | (43.3–44.3) |  | 38.9 | (38.5–39.3) |  | 39.6 | (39.3–40) |  | 44.3 | (43.6–45) |  | 50.3 | (49.7–50.8) |
| *Health Status* |  |  |  |  |  |  |  |  |  |  |  |  |  |  |
| Very Good | 22.1 | (21.4–22.8) |  | 20.3 | (19.7–20.9) |  | 18.5 | (18.1–18.9) |  | 12.7 | (12.1–13.3) |  | 15.5 | (14.9–16) |
| Good | 49.1 | (48.5–49.7) |  | 51.7 | (51.2–52.2) |  | 52.7 | (52.3–53.1) |  | 55.2 | (54.5–55.9) |  | 52.1 | (51.5–52.7) |
| Regular | 23.3 | (23–23.7) |  | 23.1 | (22.8–23.5) |  | 23.6 | (23.3–23.9) |  | 26.5 | (25.9–27.1) |  | 26.8 | (26.3–27.3) |
| Poor | 4.5 | (4.4–4.7) |  | 4 | (3.9–4.2) |  | 4.1 | (4–4.2) |  | 4.6 | (4.3–4.9) |  | 4.5 | (4.3–4.7) |
| Very Poor | 0.9 | (0.8–1) |  | 0.8 | (0.8–0.9) |  | 1 | (1–1.1) |  | 1 | (0.9–1.1) |  | 1.1 | (1–1.3) |

*Sources:* PNAD 1998, 2003, and 2008, PNS 2013 and 2019, and the authors’ calculations.

Figure A1: Prevalence of health care utilisation variables by socioeconomic status

1. Any doctor visit in the last year (2) Hospitalisation in the last year (excl. deliveries)

1. Any surgery in the last year (4) Use of Pap smear in the last 3 years

1. Use of mammograms in the last 2 years

*Sources:* PNAD 1998, 2003, and 2008, PNS 2013 and 2019, and the authors’ calculations.

Figure A2. Concentration curves for health care utilisation variables

1. Any doctor visit in the last year (2) Hospitalisation in the last year (excl. deliveries)

(3) Any surgery in the last year (4) Use of Pap smear in the last 3 years

1. Use of mammograms in the last 2 years

*Sources:* PNAD 1998, 2003, and 2008, PNS 2013 and 2019, and the authors’ calculations.

Table A3: Erreygers-corrected CInds for hospitalization variables.

|  | **1998** | |  | **2003** | |  | **2008** | |  | **2013** | |  | **2019** | |
| --- | --- | --- | --- | --- | --- | --- | --- | --- | --- | --- | --- | --- | --- | --- |
| **Variables** | CInd | 95% CI |  | CInd | 95% CI |  | CInd | 95% CI |  | CInd | 95% CI |  | CInd | 95% CI |
| **Hospitalization in the last year** |  |  |  |  |  |  |  |  |  |  |  |  |  |  |
| - All admissions | -0.023*** | (-0.026– -0.019) |  | -0.013*** | (-0.016– -0.010) |  | -0.011*** | (-0.014– -0.008) |  | -0.006 | (-0.014–0.001) |  | 0.001 | (-0.007–0.008) |
| - Excluding deliveries | -0.004*** | (-0.007– -0.001) |  | 0.004*** | (0.001–0.007) |  | 0.003* | (-0.000–0.005) |  | 0.003 | (-0.005–0.010) |  | 0.010*** | (0.004–0.016) |
| - Deliveries only | -0.019*** | (-0.020– -0.017) |  | -0.017*** | (-0.018– -0.015) |  | -0.014*** | (-0.015– -0.012) |  | -0.009*** | (-0.012– -0.006) |  | -0.009*** | (-0.012– -0.006) |

*Notes*: The findings presented include the Erreygers-corrected concentration indices (CInds) and 95% confidence intervals (CIs) for all outcome variables associated with utilization of hospital care. The values presented are parameter estimates of the concentration index (CInd) ranging from -1 (perfect pro-poor inequality) to +1 (perfect pro-rich inequality). The associated *p*-value helps to determine whether the association also exists in the larger population. Total sample sizes (***N***) for each outcome-year are presented in Table A1. **p*<0.1 ***p*<0.05 ****p*<0.01

*Sources:* PNAD 1998, 2003, and 2008, PNS 2013 and 2019, and the authors’ calculations.

Table A4. Decomposition of the CInds reveals factors that contribute to the utilisation of healthcare services (2019).

|  | Any doctor visit  in the last year | |  | Hospitalization  in the last year  (excl. deliveries) | |  | Any surgery  in the last year | |  | Use of pap smear  in the last 3 years (women 25-59) | |  | Use of mammogram in the last 2 years (women 50-69) | |
| --- | --- | --- | --- | --- | --- | --- | --- | --- | --- | --- | --- | --- | --- | --- |
|  | CInd | Contrib. % |  | CInd | Contrib. % |  | CInd | Contrib.  % |  | CInd | Contrib.  % |  | CInd | Contrib. % |
| **PREDISPOSING FACTORS** |  |  |  |  |  |  |  |  |  |  |  |  |  |  |
| *Female (omitted: Male)* | -0.024*** | -4.35% |  | -0.024*** | 2.96% |  | -0.024*** | 0.87% |  | 0.000*** | 0.00% |  | -0.000*** | 0.00% |
| *Age (omitted: 18-24)* |  |  |  |  |  |  |  |  |  |  |  |  |  |  |
| 25-34 | -0.083*** | 0.42% |  | -0.083*** | -5.50% |  | -0.083*** | -3.66% |  | 0 | 0.00% |  | 0 | 0.00% |
| 35-44 | -0.063*** | 0.54% |  | -0.063*** | -8.76% |  | -0.063*** | -5.90% |  | -0.076*** | 0.93% |  | 0 | 0.00% |
| 45-54 | 0.032*** | -0.06% |  | 0.032*** | 4.12% |  | 0.032*** | 3.03% |  | -0.056*** | -2.30% |  | 0 | 0.00% |
| 55-64 | 0.103*** | 0.24% |  | 0.103*** | 4.92% |  | 0.103*** | 4.46% |  | 0.070*** | 2.43% |  | -0.052*** | -1.88% |
| 65 or older | 0.171*** | 3.19% |  | 0.171*** | 18.53% |  | 0.171*** | 6.47% |  | 0.115*** | 0.00% |  | 0.007 | 0.15% |
| *Race (omitted: White)* |  |  |  |  |  |  |  |  |  |  |  |  |  |  |
| Black | -0.152*** | 0.07% |  | -0.152*** | 3.31% |  | -0.152*** | 2.62% |  | -0.146*** | -1.22% |  | -0.157*** | -0.61% |
| Asian | 0.180*** | 0.07% |  | 0.180*** | -0.85% |  | 0.180*** | -1.07% |  | 0.181** | 0.22% |  | 0.041 | 0.09% |
| Browns/Mixed | -0.163*** | 1.34% |  | -0.163*** | -13.75% |  | -0.163*** | -0.08% |  | -0.165*** | -2.20% |  | -0.166*** | -2.74% |
| Indigenous | -0.204*** | 0.02% |  | -0.204*** | 0.83% |  | -0.204*** | 0.42% |  | -0.196*** | -0.09% |  | -0.265** | 0.03% |
| *Education (omitted: None)* |  |  |  |  |  |  |  |  |  |  |  |  |  |  |
| Basic incomplete | -0.185*** | -0.57% |  | -0.185*** | 26.81% |  | -0.185*** | 0.33% |  | -0.285*** | -10.82% |  | -0.224*** | -4.48% |
| Basic complete | -0.113*** | -0.15% |  | -0.113*** | 3.59% |  | -0.113*** | 0.28% |  | -0.150*** | -2.86% |  | -0.023 | -0.04% |
| Secondary incomplete | -0.255*** | -0.14% |  | -0.255*** | 9.83% |  | -0.255*** | -1.83% |  | -0.241*** | -3.42% |  | -0.042 | -0.21% |
| Secondary complete | -0.015*** | -0.53% |  | -0.015*** | 1.73% |  | -0.015*** | -0.48% |  | -0.034*** | -3.77% |  | 0.102*** | 2.73% |
| Higher incomplete | 0.269*** | 0.76% |  | 0.269*** | -8.59% |  | 0.269*** | 0.38% |  | 0.204*** | 2.48% |  | 0.415*** | 0.16% |
| Higher complete | 0.536*** | 12.78% |  | 0.536*** | -41.04% |  | 0.536*** | 7.49% |  | 0.517*** | 44.57% |  | 0.575*** | 16.42% |
| *Employment status (omitted: Inactive)* |  |  |  |  |  |  |  |  |  |  |  |  |  |  |
| Inactive |  |  |  |  |  |  |  |  |  |  |  |  |  |  |
| Unemployed | -0.462*** | 3.71% |  | -0.462*** | 10.96% |  | -0.462*** | 1.98% |  | -0.365*** | -1.46% |  | -0.304*** | -0.57% |
| Employed | 0.088*** | -4.81% |  | 0.088*** | -43.24% |  | 0.088*** | -13.44% |  | 0.145*** | 6.87% |  | 0.148*** | 1.67% |
| *Presence of elderly in the HH* | 0.115*** | -0.49% |  | 0.115*** | -7.50% |  | 0.115*** | -0.90% |  | 0.109*** | -3.18% |  | 0.048*** | -0.25% |
| *Presence of a child in the HH* | -0.317*** | -2.06% |  | -0.317*** | 14.73% |  | -0.317*** | -0.86% |  | -0.241*** | -4.19% |  | -0.422*** | 1.08% |
| **ENABLING FACTORS** |  |  |  |  |  |  |  |  |  |  |  |  |  |  |
| *Income quintile (omitted: Poorest quintiles)* |  |  |  |  |  |  |  |  |  |  |  |  |  |  |
| Poor quintile | -0.416*** | -3.81% |  | -0.416*** | -4.17% |  | -0.416*** | -8.69% |  | -0.319*** | -3.92% |  | -0.549*** | -3.12% |
| Average quintile | -0.009* | -0.12% |  | -0.009* | -0.83% |  | -0.009* | -0.60% |  | 0.072*** | 1.26% |  | -0.142*** | -3.10% |
| Rich quintile | 0.396*** | 9.95% |  | 0.396*** | 13.25% |  | 0.396*** | 25.97% |  | 0.439*** | 11.58% |  | 0.306*** | 7.11% |
| Richest quintile | 0.784*** | 22.93% |  | 0.784*** | 56.99% |  | 0.784*** | 49.93% |  | 0.805*** | 28.13% |  | 0.757*** | 35.00% |
| *Area of residence (omitted: Rural)* |  |  |  |  |  |  |  |  |  |  |  |  |  |  |
| Urban | 0.047*** | 2.68% |  | 0.047*** | 0.70% |  | 0.047*** | 0.69% |  | 0.045*** | -0.92% |  | 0.035*** | 2.27% |
| *Region (omitted: South)* |  |  |  |  |  |  |  |  |  |  |  |  |  |  |
| North | -0.281*** | 3.11% |  | -0.281*** | 12.51% |  | -0.281*** | 3.31% |  | -0.256*** | 1.59% |  | -0.297*** | 2.96% |
| North-East | -0.286*** | 8.66% |  | -0.286*** | 51.86% |  | -0.286*** | 12.77% |  | -0.294*** | 8.51% |  | -0.282*** | 3.04% |
| South-East | 0.127*** | 3.52% |  | 0.127*** | -8.80% |  | 0.127*** | 3.59% |  | 0.124*** | -1.17% |  | 0.122*** | 3.48% |
| Midwest | 0.098*** | -0.68% |  | 0.098*** | 3.25% |  | 0.098*** | -0.31% |  | 0.111*** | -1.47% |  | 0.076*** | -0.26% |
| *Private Health Insurance* | 0.407*** | 50.59% |  | 0.407*** | 144.06% |  | 0.407*** | 39.64% |  | 0.411*** | 33.74% |  | 0.428*** | 34.23% |
| **NEED FACTORS** |  |  |  |  |  |  |  |  |  |  |  |  |  |  |
| *Presence of at least one NCD* | 0.035*** | 7.42% |  | 0.035*** | 29.42% |  | 0.035*** | 7.64% |  | -0.005 | 0.00% |  | -0.014*** | -1.43% |
| *Health Status (omitted: Very Good)* |  |  |  |  |  |  |  |  |  |  |  |  |  |  |
| Good | 0.020*** | 1.20% |  | 0.020*** | 7.04% |  | 0.020*** | 1.10% |  | 0.047*** | -0.84% |  | 0.127*** | -1.49% |
| Regular | -0.121*** | -10.89% |  | -0.121*** | -76.18% |  | -0.121*** | -15.37% |  | -0.181*** | 1.39% |  | -0.149*** | 3.51% |
| Poor | -0.216*** | -5.89% |  | -0.216*** | -40.02% |  | -0.216*** | -7.26% |  | -0.293*** | 1.50% |  | -0.312*** | 1.75% |
| Very Poor | -0.210*** | -1.31% |  | -0.210*** | -12.80% |  | -0.210*** | -1.42% |  | -0.285*** | 0.72% |  | -0.247*** | 0.89% |
|  |  |  |  |  |  |  |  |  |  |  |  |  |  |  |
| **Number of observations** | 83,927 |  |  | 83,927 |  |  | 83,927 |  |  | 28,778 |  |  | 14,518 |  |
| **Total contribution** | 97.38% |  |  | 149.36% |  |  | 111.08% |  |  | 102.10% |  |  | 96.41% |  |

*Notes:* The findings presented include the Erreygers-corrected concentration indices and the contribution to CInds for the factors that contribute to unmet needs for health care services and medications. **p*<0.1. ***p*<0.05. ****p*<0.01. HH stands for household, NCD stands for non–communicable diseases.

*Sources:* PNAD 1998, 2003, and 2008, PNS 2013 and 2019, and the authors’ calculations.

Figure A3: Prevalence of health care utilisation variables by region of residence.

1. Any doctor visit in the last year (2) Hospitalisation in the last year (excl. deliveries)

(3) Any surgery in the last year (4) Use of Pap smear in the last 3 years

(5) Use of mammograms in the last 2 years

*Sources:* PNAD 1998, 2003, and 2008, PNS 2013 and 2019, and the authors’ calculations.

Figure A4: Prevalence of health care utilisation variables by socioeconomic status and region of residence.

**(1) Any doctor visit in the last year**

(a) Brazil (b) South

(c) North (d) North-East

(e) South-East (f) Midwest

*Sources:* PNAD 1998, 2003, and 2008, PNS 2013 and 2019, and the authors’ calculations.

**(2) Hospitalization in the last year (excl. deliveries)**

(a) Brazil (b) South

(c) North (d) North-East

(e) South-East (f) Midwest

*Sources:* PNAD 1998, 2003, and 2008, PNS 2013 and 2019, and the authors’ calculations.

**(3) Any surgery in the last year**

(a) Brazil (b) South

(c) North (d) North-East

(e) South-East (f) Midwest

*Sources:* PNAD 1998, 2003, and 2008, PNS 2013 and 2019, and the authors’ calculations.

**(4) Use of Pap smear in the last 3 years**

(a) Brazil (b) South

(c) North (d) North-East

(e) South-East (f) Midwest

*Sources:* PNAD 1998, 2003, and 2008, PNS 2013 and 2019, and the authors’ calculations.

**(5) Use of mammograms in the last 2 years**

(a) Brazil (b) South

(c) North (d) North-East

(e) South-East (f) Midwest

*Sources:* PNAD 1998, 2003, and 2008, PNS 2013 and 2019, and the authors’ calculations.

Figure A5: Decomposition of the CInds reveals factors that contribute to inequality in healthcare utilization variables by region (2019).

**Any doctor visit in the last year**

**Hospitalisation in the last year (excl. deliveries)**

**Any surgery in the last year**

**Use of Pap smear in the last 3 years (women 25-59)**

**Use of mammogram in the last 2 years (women 50-69)**

*Notes*: Regions marked with a star have non-statistically significant CInds. HH stands for household, NCD stands for non–communicable diseases.

*Sources:* PNAD 1998, 2003, and 2008, PNS 2013 and 2019, and the authors’ calculation.

# *Appendix 3. Robustness tests*

*R1. Robustness of main results to the inclusion of new covariates in the model related to income inequality, poverty, and availability of healthcare services.*

Figure R1.1 Decomposition of the CInds reveals factors that contribute to inequality in health care utilisation variables using additional covariates, including poverty (state-level), availability of mammographers, and HHs registered in the FHS (2019).


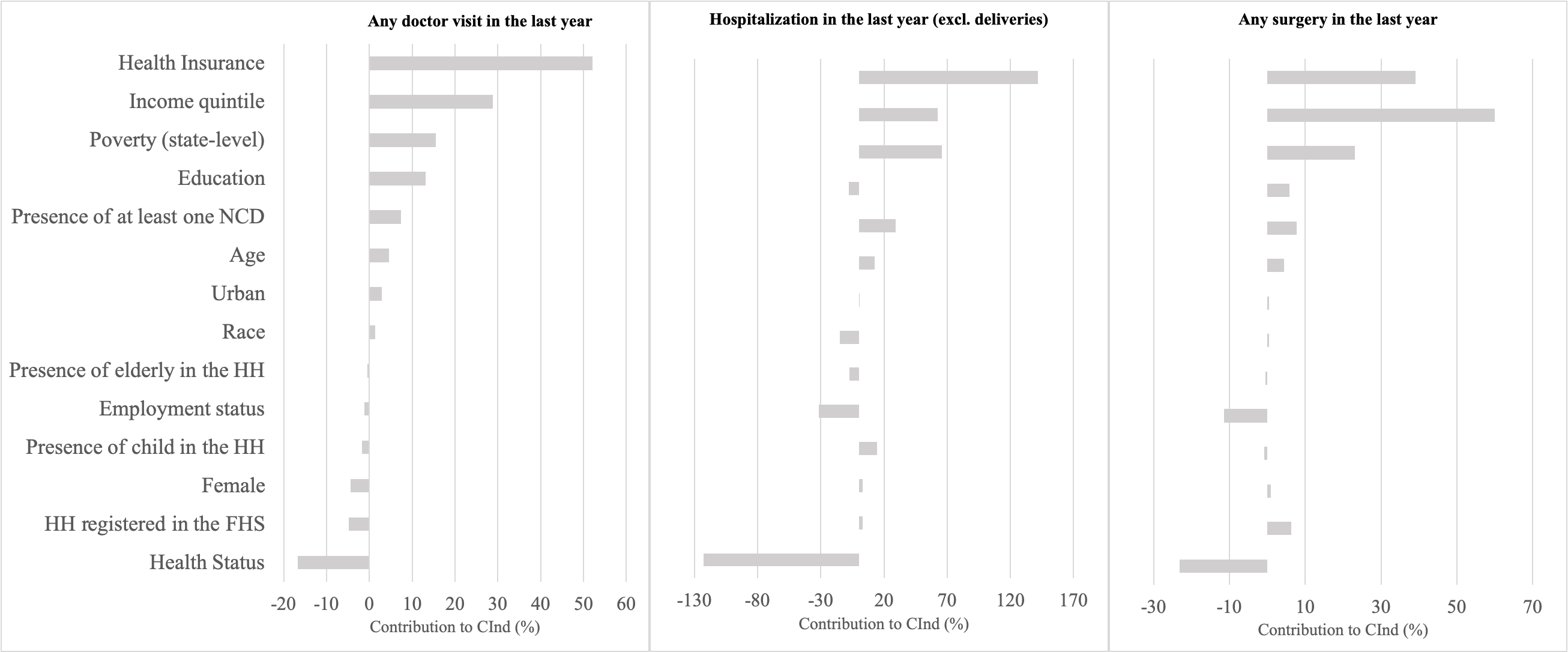


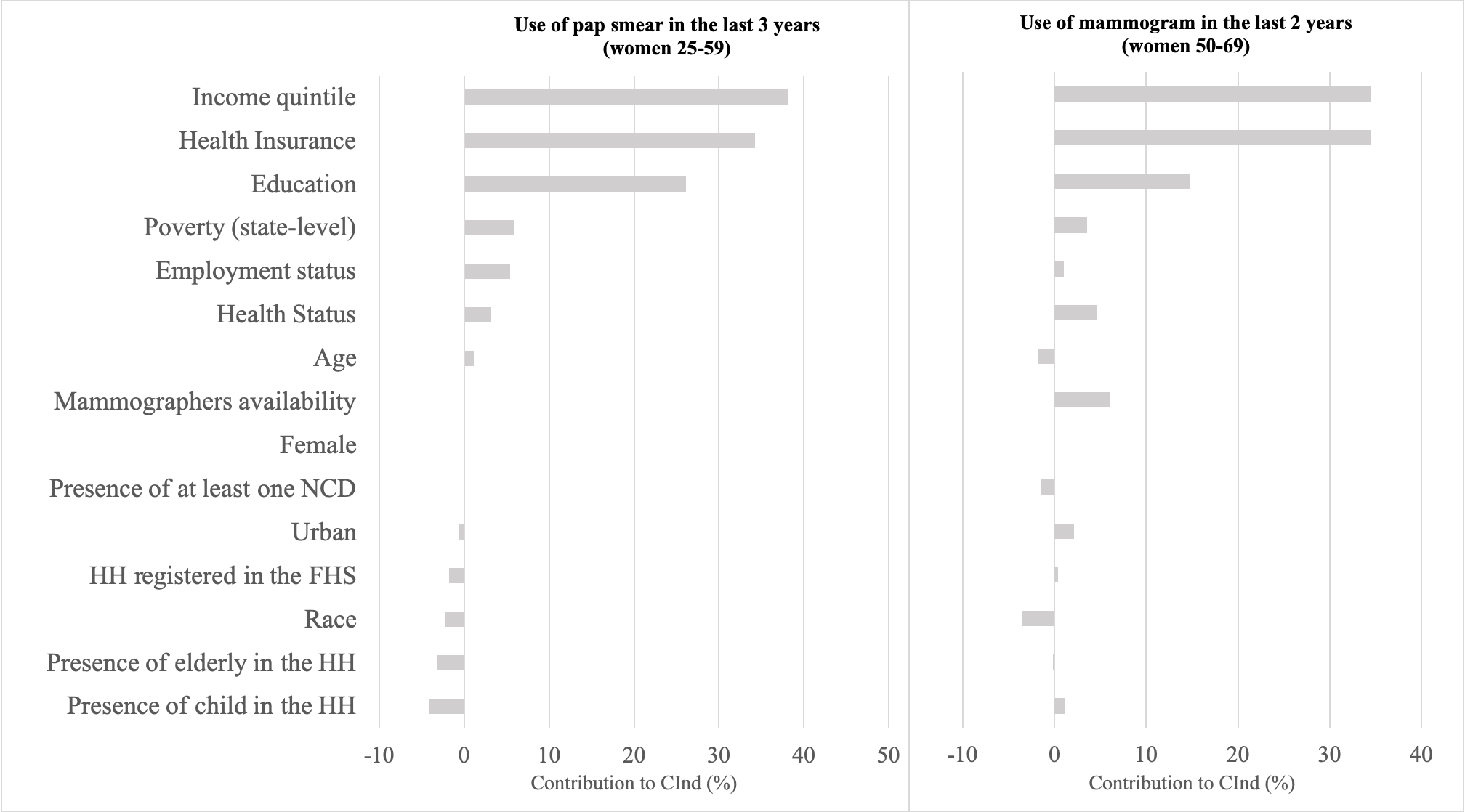


*Sources:* PNS 2019 and the authors’ calculations. Abbreviations: HH, household; NCD, non–communicable diseases; FHS, Family Health Strategy. The following variables were included as covariates: (1) Poverty (state-level): percentage of the total population in the state of residence in basic or extreme poverty. Poverty is defined as income per capita <70 reais and extreme poverty between 70.01 and 140 reais (data source: Cadastro Único Dataset - Families/Persons by per capita income range). (2) Availability of mammographers was defined as the number of mammographers per 1000 inhabitants calculated at the state level (data source: CNES – Datasus). (3) HHs registered in the FHS are those households covered by the Family Health Strategy (data source: PNAD and PNS). The covariates generated at the state level have substituted for regional fixed effects in the main specification of the model.

Figure R1.2 Decomposition of the CInds reveals factors that contribute to inequality in health care utilisation variables using new covariates: inequality (state-level), availability of mammographers, and HHs registered in the FHS (2019).


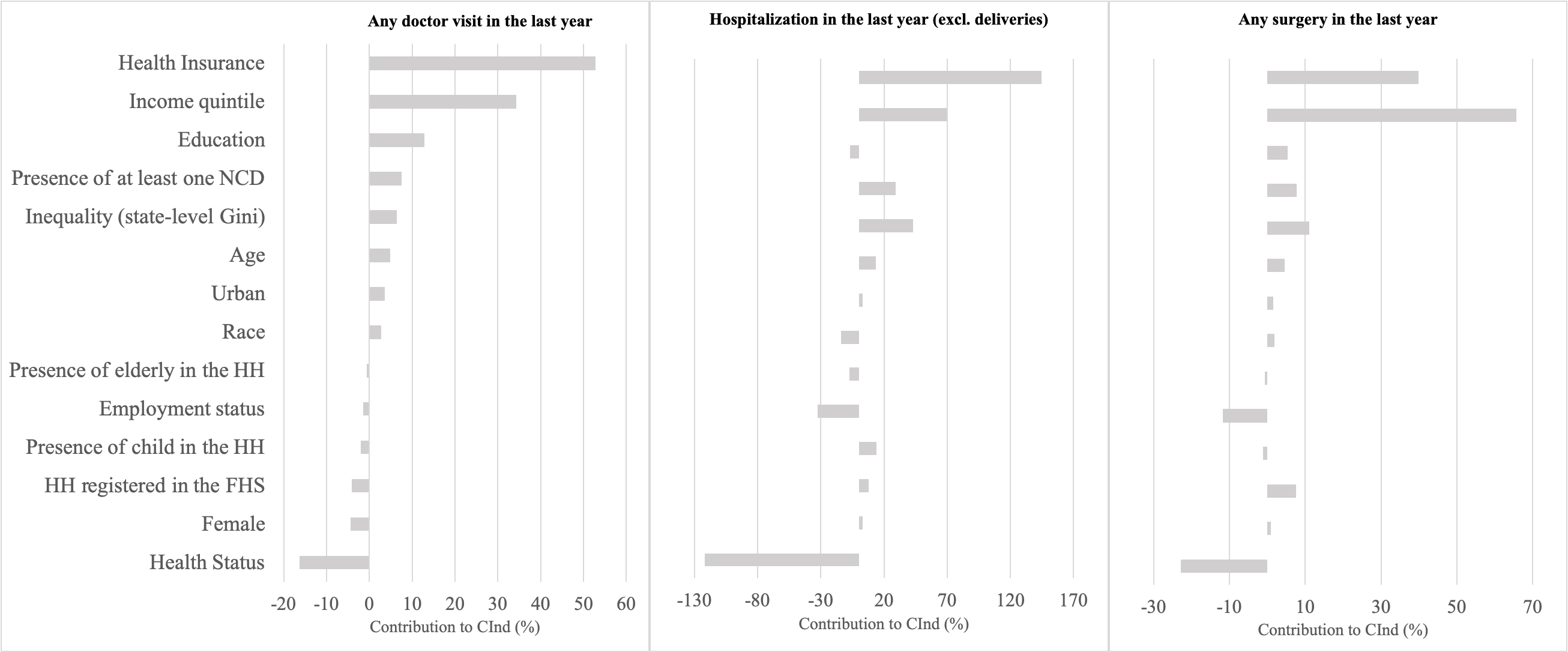


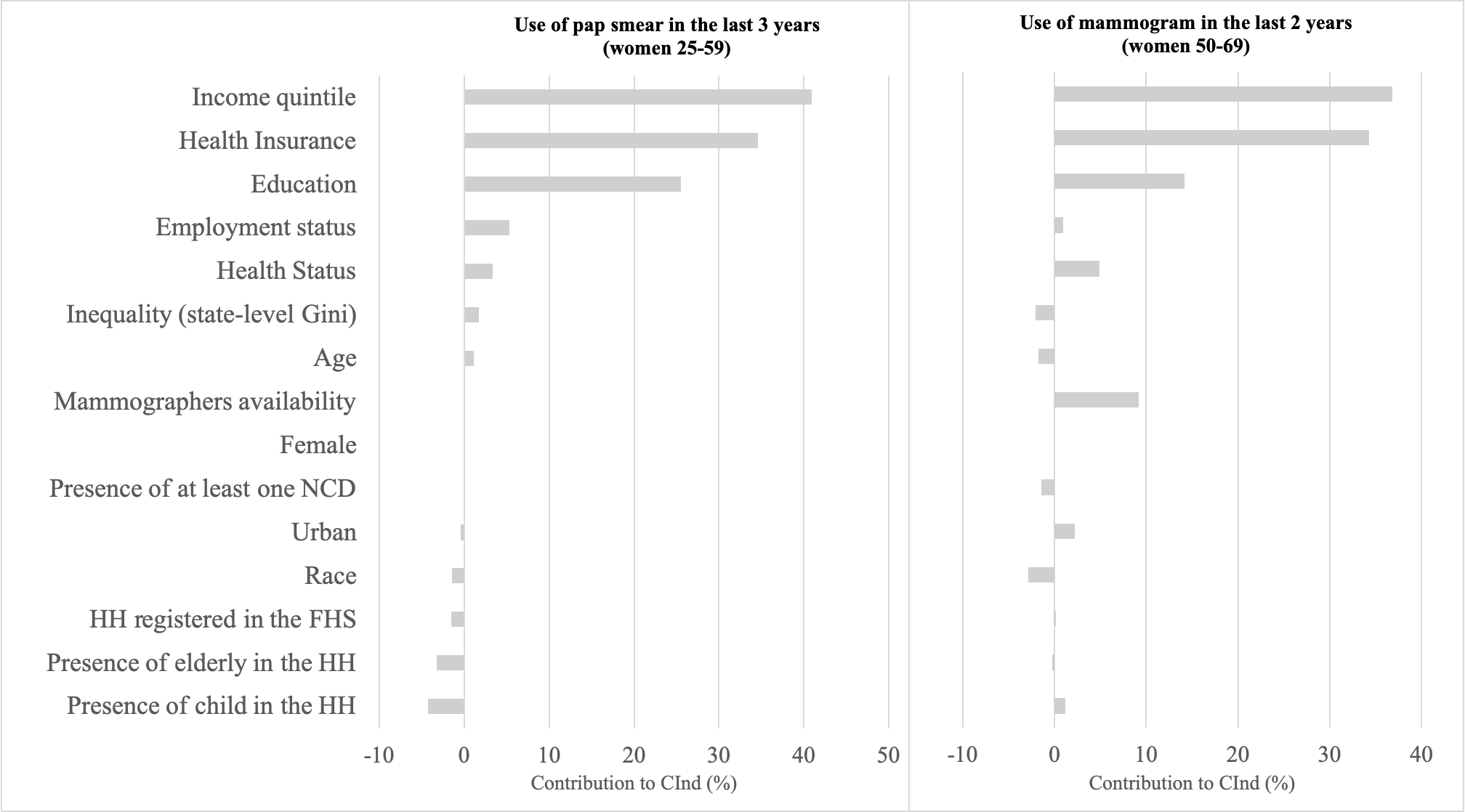


*Sources:* PNS 2019 and the authors’ calculations. Abbreviations: HH, household; NCD, non–communicable diseases; FHS, Family Health Strategy. The following variables were included as covariates: (1) Inequality (state-level Gini), defined as the value of the Gini coefficient of household income per capita of the resident population in 2010, calculated at the state level (data source: IBGE/Censo Demográfico 2010); (2) Availability of mammographers was defined as the number of mammographers available per 1000 inhabitants, calculated at the state level (data source: CNES – Datasus). (3) HHs registered in the FHS indicate whether a given household is covered by the Family Health Strategy (data source: PNAD and PNS). The covariates generated at the state level have substituted for region fixed-effects in the main specification of the model.

*R2. Robustness of main results to the method.*

Table R2.1 Wagstaff-corrected CInds for health care utilisation variables.

|  | **1998** | |  | **2003** | |  | **2008** | |  | **2013** | |  | **2019** | |
| --- | --- | --- | --- | --- | --- | --- | --- | --- | --- | --- | --- | --- | --- | --- |
| **Variables** | CInd | 95% CI |  | CInd | 95% CI |  | CInd | 95% CI |  | CInd | 95% CI |  | CInd | 95% CI |
| Any doctor visit  in the past year | 0.124*** | (0.114–0.133) |  | 0.148*** | (0.140–0.157) |  | 0.125*** | (0.118–0.132) |  | 0.181*** | (0.163–0.199) |  | 0.189*** | (0.172–0.207) |
| Hospitalisation  in the past year (excluding labour and delivery) | -0.017*** | (-0.029–-0.004) |  | 0.016*** | (0.005–0.027) |  | 0.011* | (-0.000–0.022) |  | 0.011 | (-0.020–0.042) |  | 0.039*** | (0.014–0.063) |
| Any surgery  in the past year | 0.141*** | (0.121–0.162) |  | 0.154*** | (0.137–0.172) |  | 0.140*** | (0.123–0.158) |  | 0.112*** | (0.063–0.161) |  | 0.133*** | (0.097–0.169) |
| Use of Pap smears  in the past three years (women aged 25–59 years) |  |  |  | 0.263*** | (0.250–0.275) |  | 0.223*** | (0.209–0.236) |  | 0.243*** | (0.210–0.276) |  | 0.237*** | (0.210–0.264) |
| Use of mammograms in the past two years (women aged 50–69 years) |  |  |  | 0.412*** | (0.398–0.427) |  | 0.351*** | (0.338–0.364) |  | 0.362*** | (0.324–0.399) |  | 0.274*** | (0.244–0.304) |

*Notes*: Wagstaff-corrected Concentration Indices (CInds) and 95% confidence intervals (CIs) are presented for all outcome variables associated with health care services utilisation. The values presented are parameter estimates of the concentration index (CInd) ranging from -1 (perfect pro-poor inequality) to +1 (perfect pro-rich inequality). The associated *p*-value helps to determine whether the association also exists in the larger population. Total sample sizes (***N***) for each outcome-year are presented in Table 1. The outcome variable ‘hospitalisations in the past year’ excludes hospital admissions for labour and delivery (vaginal birth and C-sections); **p*<0.1; ***p*<0.05; ****p*<0.01

*Sources:* PNAD 1998, 2003, and 2008, PNS 2013 and 2019, and the authors’ calculations.

*R3. Robustness of main results to new preventive care outcomes.*

Table R3.1 Summary statistics for health care utilisation variables related to preventive care (new).

|  |  | **2013** | | |  | **2019** | | |
| --- | --- | --- | --- | --- | --- | --- | --- | --- |
| **Variable** |  | n | % | 95% CI |  | n | % | 95% CI |
| Blood pressure test in the past two years (adults 18 years or older) |  | 51,132 | 89.4 | (88.9–89.8) |  | 76,709 | 91.4 | (91.1–91.8) |
| Cholesterol test in the past five years (men 35 years or older) |  | 11,273 | 72.6 | (71.2–74) |  | 22,588 | 80.0 | (79–80.9) |
| Blood sugar test in the past three years (adults 45 years or older) |  | 20,595 | 84.4 | (83.7–85.2) |  | 39,334 | 88.9 | (88.4–89.4) |

*Notes*: The findings presented include the mean prevalence for all outcome variables, including the number of observations (n), percentage (%), and 95% confidence intervals (CIs). The total sample of each outcome variable includes only the corresponding eligible population based on guidelines from the Brazilian Ministry of Health^[1-3]^. The number of observations for adults 18 years of age or older include n = 57,195 (2013), and n = 83,927 (2019). The number of observations for men 35 years of age or older include n = 15,528 (2013), and n = 28,235 (2019). The number of observations for adults 45 years of age or older include n = 24,402 (2013), and n = 44,245 (2019).

*Sources:* PNS 2013 and 2019, and the authors’ calculations.

Table R3.2 Erreygers-corrected CInds for health care utilisation variables related to preventive care (new).

|  |  | **2013** | |  | **2019** | |
| --- | --- | --- | --- | --- | --- | --- |
| **Variables** |  | CInd | 95% CI |  | CInd | 95% CI |
| Blood pressure test in the past two years (adults 18 years or older) |  | 0.090*** | (0.080–0.100) |  | 0.083*** | (0.075–0.091) |
| Cholesterol test in the past five years (men 35 years or older) |  | 0.209*** | (0.180–0.238) |  | 0.186*** | (0.165–0.206) |
| Blood sugar test in the past three years (adults 45 years or older) |  | 0.150*** | (0.134–0.167) |  | 0.110*** | (0.099–0.121) |

*Notes*: Erreygers-corrected Concentration Indices (CInds) and 95% confidence intervals (CIs) are presented for all outcome variables associated with health care services utilisation. The presented values are parameter estimates of the concentration index. The values presented are parameter estimates of the concentration index (CInd) ranging from -1 (perfect pro-poor inequality) to +1 (perfect pro-rich inequality). The associated *p*-value helps to determine whether the association also exists in the larger population. Total sample sizes (***N***) for each outcome-year are presented in Table EXTRA 2. **p*<0.1; ***p*<0.05; ****p*<0.01.

*Sources:* PNS 2013 and 2019, and the authors’ calculations.

Figure R3.1 Decomposition of the CInds reveals factors that contribute to inequality in healthcare utilization variables related to preventive care (2019).


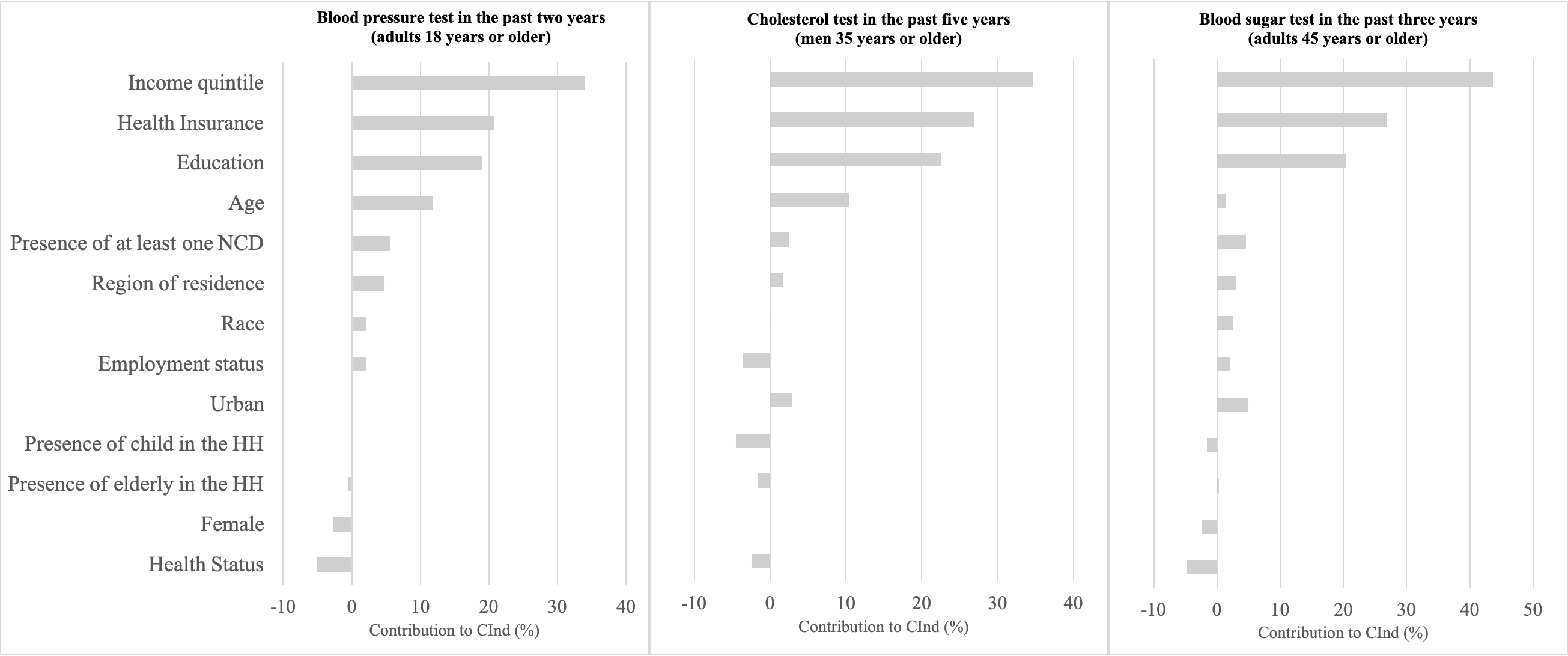


*Sources:* PNS 2019, and the authors’ calculations. HH stands for household, NCD stands for non–communicable diseases.

References

1. Brasil (2010). Ministério da Saúde. Secretaria de Atenção à Saúde. Departamento de Atenção Básica. Rastreamento. Brasília: Ministério da Saúde. 95 p.: il. – (Série A. Normas e Manuais Técnicos) (Cadernos de Atenção Primária, n. 29)
2. Brasil (2013). Ministério da Saúde. Secretaria de Atenção à Saúde. Departamento de Atenção Básica. Estratégias para o cuidado da pessoa com doença crônica: hipertensão arterial sistêmica. Brasília: Ministério da Saúde. 128 p.: il. (Cadernos de Atenção Básica, n. 37)
3. Brasil (2013). Ministério da Saúde. Secretaria de Atenção à Saúde. Departamento de Atenção Básica. Estratégias para o cuidado da pessoa com doença crônica: diabetes mellitus. Brasília: Ministério da Saúde. 160 p.: il. (Cadernos de Atenção Básica, n. 36)

1. The use of income quintile variable in the decomposition of CInd will avoid the bias that would be generated by omitting the variable in the regressions. For instance, Wagstaff et al. (2003) demonstrated that consumption–related inequalities in child malnutrition could be explained by the direct impact of household consumption and by community–level correlates of both malnutrition and consumption. [↑](#footnote-ref-1)
2. Region of residence captures factors related to supply of hospital beds and physicians in the community where the individuals live. [↑](#footnote-ref-2)
3. The question about self–reported prevalence of chronic disease has changed slightly over time to incorporate the physician diagnostic. The question “Do you have [disease]?” in 1998/2003 has since changed to “Has any doctor or health professional said that you have [disease]?” in 2008 and “Has any doctor ever given you the diagnosis of [disease]?” in 2013/2019. [↑](#footnote-ref-3)
